# Perturb-seq reveals TCF7 as a transcriptional link between MAPK- and Wnt-driven gene expression

**DOI:** 10.1093/nar/gkag718

**Published:** 2026-07-29

**Authors:** Ghanem El Kassem, Anja Sieber, Bertram Klinger, Florian Uhlitz, David Steinbrecht, Mirjam van Bentum, Shawez Khan, Jasmine Hillmer, Jennifer von Schlichting, Reinhold Schäfer, Nils Blüthgen, Michael Boettcher

**Affiliations:** Institute of Molecular Medicine, Section for Molecular Medicine of Signal Transduction, Faculty of Medicine, Martin-Luther-University Halle-Wittenberg, 06120 Halle (Saale), Germany; Institute of Pathology, Charité - Universitätsmedizin Berlin, Charitéplatz 1, 10117 Berlin, Germany; Institute of Pathology, Charité - Universitätsmedizin Berlin, Charitéplatz 1, 10117 Berlin, Germany; Institute of Pathology, Charité - Universitätsmedizin Berlin, Charitéplatz 1, 10117 Berlin, Germany; Institute of Pathology, Charité - Universitätsmedizin Berlin, Charitéplatz 1, 10117 Berlin, Germany; Institut für Biologie, Humboldt Universität zu Berlin, Haus 18, Philippstr. 13, 10115 Berlin; Institute of Pathology, Charité - Universitätsmedizin Berlin, Charitéplatz 1, 10117 Berlin, Germany; Max Delbrück Center for Molecular Medicine, Robert-Rössle-Straße 10, 13125 Berlin, Germany; Institute of Molecular Medicine, Section for Molecular Medicine of Signal Transduction, Faculty of Medicine, Martin-Luther-University Halle-Wittenberg, 06120 Halle (Saale), Germany; Institute of Molecular Medicine, Section for Molecular Medicine of Signal Transduction, Faculty of Medicine, Martin-Luther-University Halle-Wittenberg, 06120 Halle (Saale), Germany; Institute of Pathology, Charité - Universitätsmedizin Berlin, Charitéplatz 1, 10117 Berlin, Germany; Comprehensive Cancer Center, Charité - Universitätsmedizin Berlin, Charitéplatz 1, 10117 Berlin, Germany; German Consortium for Translational Cancer Research (DKTK), German Cancer Research Center (DKFZ), Im Neuenheimer Feld 280, 69120 Heidelberg, Germany; Institute of Pathology, Charité - Universitätsmedizin Berlin, Charitéplatz 1, 10117 Berlin, Germany; Institut für Biologie, Humboldt Universität zu Berlin, Haus 18, Philippstr. 13, 10115 Berlin; German Consortium for Translational Cancer Research (DKTK), German Cancer Research Center (DKFZ), Im Neuenheimer Feld 280, 69120 Heidelberg, Germany; Institute of Molecular Medicine, Section for Molecular Medicine of Signal Transduction, Faculty of Medicine, Martin-Luther-University Halle-Wittenberg, 06120 Halle (Saale), Germany

## Abstract

The mitogen-activated protein kinase (MAPK) pathway is a central signaling cascade whose dysregulation contributes to numerous diseases. While its upstream regulation is well studied, the mechanisms by which MAPK activation leads to diverse transcriptional outcomes remain incompletely understood. To address this shortcoming, we mapped the target gene sets controlled by 22 RAF-inducible transcription factors using targeted Perturb-seq and integrated these data with time-resolved transcriptional profiling. Network reconstruction revealed a topology dominated by two central hubs, EGR1 and FOS, which co-regulate partially overlapping target gene sets. In addition, we uncovered a positive feedback loop between EGR1, a canonical RAF–MAPK effector, and TCF7, a transcription factor typically linked to Wnt signaling. Through this interaction, TCF7 emerges as a transcriptional link that integrates MAPK and Wnt pathway inputs. Together, these findings define the architecture of RAF–MAPK-driven transcriptional regulation and demonstrate how cross-talk between oncogenic signaling pathways can be encoded in transcriptional networks.

## Graphical Abstract

**Graphical Abstract:**
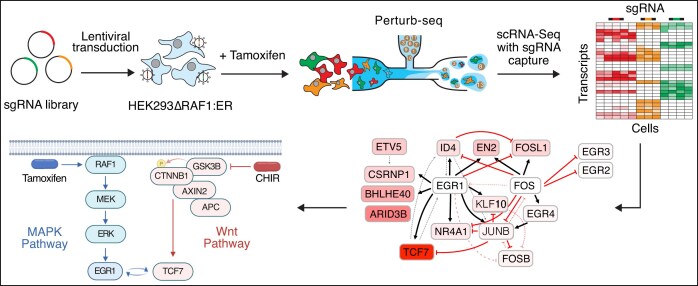

## Introduction

The mitogen-activated protein kinase (MAPK) signaling cascade is a fundamental and evolutionarily conserved pathway that regulates proliferation, differentiation, and survival [1]. Activated by diverse external cues—including growth factors, hormones, and stress—MAPK transmits signals from the cell surface to the nucleus, where MAPKs phosphorylate transcription factors and initiate defined transcriptional programs. This adaptability allows cells to translate a broad spectrum of stimuli into context-specific responses.

The MAPK pathway has long served as a paradigm for studying signal-induced transcriptional programs [2]. Early transcriptome studies after stimulation of the pathway led to the concepts of immediate-early and delayed primary genes that are activated by pre-existing transcription factors [3] and share regulatory motifs in their promoters [4, 5]. Many immediate-early genes are transcription factors, which then induce secondary targets. Interestingly, delayed primary genes often encode negative feedback regulators [6, 7, 8], and the timing of primary response genes is strongly determined by messenger RNA (mRNA) half-lives [9]. While the kinetics of the transcriptome response to MAPK activation has been characterized in quantitative depth [3, 6, 8, 9], the wiring of how immediate-early transcription factors induce secondary response genes and the understanding of their interaction remain cryptic. A better understanding of the topology of those transcriptional networks will be crucial for understanding the coordinated regulation of oncogenic pathways.

Aberrant MAPK signaling is a hallmark of many diseases, particularly cancer [10, 11]. For example, driver Epidermal Growth Factor Receptor (EGFR) gene mutations occur in roughly 10%–35% of non-small cell lung cancers, most frequently as exon 19 deletions or the L858R substitution in exon 21 [12]. Activating mutations in Kirsten rat sarcoma virus oncogene (KRAS) that lead to aberrant activation of the MAPK signaling pathway are the most common with approximately 30% of all cancers carrying driver Ras mutations [13]. These are followed by B-Raf proto-oncogene (BRAF) mutations which account for around 8% of cancer cases, especially in melanoma and thyroid cancers [12]. Reverse engineering of the transcriptional networks downstream of MAPK signaling represents a promising approach to better understand how physiological and pathological MAPK signals are integrated into a cellular response. We have previously used systematic perturbation data and reverse engineering to elucidate a small seven-node transcription factor network downstream of Rat **s**arcoma virus (RAS)/MAPK signaling that controls transformation and different aspects of cell growth [14]. Expansion of similar approaches to larger networks has been hampered by technical challenges for a long time. However, newly developed methods that combine CRISPR-based genetic perturbation techniques with single-cell RNA-seq readout, such as Perturb-seq [15, 16], now enable simultaneous functional genetic perturbation and investigation of the resulting transcriptional response at scale.

Here, we systematically analyzed 22 RAF/MAPK-inducible transcription factors via targeted Perturb-seq (TAP-seq; [17]) to map the transcriptional programs downstream of the MAPK effector kinase RAF1. We integrated the results with time-resolved expression data to reconstruct the network topology. This analysis revealed two central hubs, EGR1 and FBJ murine osteosarcoma viral oncogene (FOS), which co-regulate overlapping and partially orthogonal target modules. Most notably, we discovered a functional transcriptional positive feedback loop between EGR1 and TCF7, a transcription factor classically linked to Wnt signaling, despite their distinct induction kinetics. Pathway-level analysis demonstrated that this transcriptional relationship enhances Wnt output, establishing TCF7 as a transcriptional link of MAPK–Wnt cross-talk. Together, these findings delineate the architecture of RAF–MAPK-driven transcriptional programs and identify a transcriptional feedback loop between EGR1 and TCF7 as a candidate mechanism linking MAPK and Wnt pathway outputs.

## Materials and methods

### Reagents

**Table 1.** Reagents. Enzymes, antibodies, kits, instruments and chemicals used in this study, with supplier, supplier location and catalog number

Reagent	Company	Location	Catalog #
**Enzymes**			
Phusion Flash High-Fidelity PCR Master Mix	Thermo Fisher Scientific	United States	F548L
Gibson Assembly Master Mix	New England Biolabs	United States	E2611L
AarI	Thermo Fisher Scientific	United States	ER1582
Titanium Taq polymerase	Takara Bio	United States	639209
KAPA HiFi HS RM	Roche	Switzerland	KK2601
ORA qPCR Green ROX L Mix	highQu	Germany	QPD0105
**Antibodies**			
CD46 Antibody, anti-human, APC, REAfinity	Miltenyi Biotec	Germany	130-130-362
**Kits**			
Illumina TruSeq mRNA Library Prep Kit v2	Illumina	United States	RS-122-2001
NucleoSpin Gel and PCR Clean-up	Macherey-Nagel	Germany	740609.250
NucleoBond Xtra Midi kit for transfection-grade plasmid DNA	Macherey-Nagel	Germany	740410.50
10x Genomics Chromium Next GEM Single Cell 3′ Reagent Kits v3.1 (Dual Index) with Feature Barcode technology for CRISPR Screening	10x Genomics	United States	1 000 268
DNeasy Blood & Tissue Kit	Qiagen	Germany	69 506
MiniSeq Mid Output Kit (300-cycles)	Illumina	United States	FC-420-1004
Quick-DNA 96 Kit	Zymo	Germany	D3012
RNeasy Mini Kit	Qiagen	Germany	74 106
QuantSeq 3′ mRNA-Seq V2	Lexogen	Austria	191.24
RevertAid H Minus First Strand cDNA Synthesis Kit	Thermo Fisher Scientific	United States	K1632
Universal KAPA Library Quantification Kit	Roche	Switzerland	07960140001
**Instruments**			
Illumina NovaSeq X	Illumina	United States	NA
Illumina HiSeq 2000	Illumina	United States	NA
Illumina MiniSeq	Illumina	United States	NA
BD FACSAria II	BD Biosciences	United States	NA
TAPE Station	Agilent	United States	NA
LightCycler^®^ 480 System	Roche	Switzerland	NA
**Chemicals**			
4-Hydroxytamoxifen	Sigma–Aldrich	Germany	H7904-5MG
CHIR99021	Sigma–Aldrich	Germany	SML1046-5MG
Puromycin dihydrochloride	Carl Roth	Germany	0240.4

See Table 1.

### Biological resources

**Table 2.** Biological resources. Cell lines, bacterial strains and plasmids used in this study, with repository, location and resource number

Reagent	Repository	Location	Resource #
**Cell lines**			
HEK293∆RAF1:ER	Derived from human embryonic kidney 293 (HEK293) cells by stable transfection of a tamoxifen‐inducible ∆RAF1:ER fusion construct; originally described in Uhlitz *et al.*, 2017.		
Lenti-X^™^ 293T Cell Line	Takara Bio	United States	632180
**Strains**			
MegaX DH10β cells	Thermo Fisher Scientific	United States	C640003
**Plasmids**			
pMB1	Addgene	United States	228363
psPAX2	Addgene	United States	12260
pMD2.G	Addgene	United States	12259

See Table 2.

### Statistical analysis

For all experiments, the number of technical and/or biological replicates is listed in the figure legends or text. Pearson correlation was used to determine the *r* values. Wald-*P*-values were adjusted using Bonferroni correction method. Statistical analyses were performed using GraphPad Prism 9 (GraphPad Software) or the R language programming environment.

### Vector maps

The all-in-one Cas9 vector pMB1 [18], containing the 10x Genomics capture sequence 1, 5′-GCTTTAAGGCCGGTCCTAGCAA-3′, in the stem-loop of the Cas9-tracr sequence [19], referred to here as pMB1-10x, was used for the Perturb-seq experiments (Table 1). The plasmid map is provided in GenBank format (Supplementary Data S1).

### HEK293∆RAF1:ER cell culture

**Figure 1.**
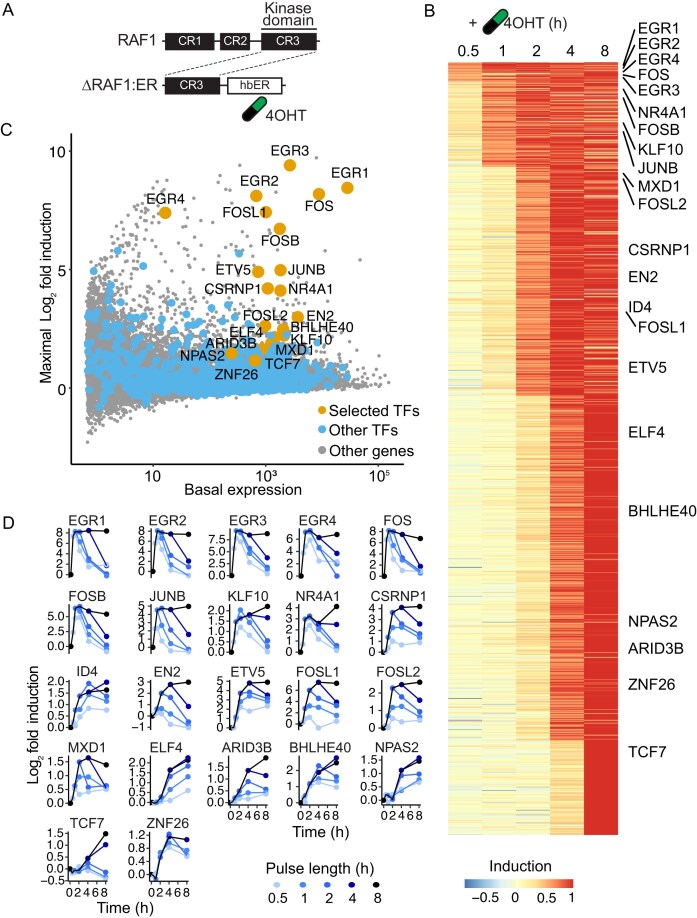
Transcriptional profiling of HEK293∆RAF1:ER cells following RAF1 induction. (**A**) Schematic structure of the 4OHT-inducible RAF1–CR3 kinase domain. (**B**) Maximum normalized log_2_ fold changes from bulk RNA-seq analysis of significantly up-regulated genes (FDR < 1%) after 0.5, 1, 2, 4 and 8 h. The 22 selected candidate TFs are indicated by their official gene symbols. (**C**) The maximal log_2_ fold expression change of the selected candidate TFs is plotted against their expression levels in non-induced HEK293∆RAF1:ER cells. Orange: Selected TFs, Blue: Other TFs, Gray: Other genes. (**D**) The time-resolved log_2_ fold expression changes of candidate TFs are shown after the indicated pulse lengths of 4OHT-mediated RAF1 induction.

HEK293∆RAF1:ER cells [20] containing a tamoxifen-inducible fusion of the kinase domain of RAF1 [21, 22] reviewed in [21, 22] were cultured in complete DMEM low glucose without phenol red supplemented with 10% fetal bovine serum (Pan Biotech) and 1% antibiotics (pen/strep) (Table 2). For the RAF1 transcriptome time-course experiment shown in Fig. 1B, cells were serum starved prior to 4OHT stimulation as described previously by Uhlitz *et al.* (2017) [9]. Lenti-X 293T cells (Takara) were cultured in complete DMEM supplemented with 10% fetal bovine serum and 1% antibiotics (pen/strep).

### Bulk RNA-sequencing time series data generation

Total RNA was extracted with TRIzol. Sequencing libraries were prepared using Illumina TruSeq mRNA Library Prep Kit v2 and sequenced on Illumina HiSeq 2000. Raw reads were processed using the snakemake-workflows rna-seq-star-deseq2 pipeline, v1.2.0, and counts were subsequently analyzed using DESeq2 in R.

### Cas9 library design

Target genes were selected based on RNA-seq data after induction of HEK293∆RAF1:ER cells with 0.5 µM 4-hydroxytamoxifen (4OHT) at different time points (Fig. 1). The single-guide RNA (sgRNA) library consisted of 4 sgRNAs per gene with 10 non-target control sgRNA and 10 safe cutter sgRNAs that cut in gene-free regions of the genome to control for potential DNA double-strand break induced side effects. The sgRNA sequences were selected from the Brunello genome wide library which was computationally optimized for high on-target activity and reduced off-target effects [23]. Four positive-control sgRNAs against the Raf-transgene were designed using CRISPick [23, 24] (Supplementary Table S1).

### Cas9 library cloning

The selected 20-nt target specific sgRNA sequences were cloned into the pMB1-10x library vector (Supplementary Data S1) by Gibson Assembly [25]. sgRNA template sequences of the format: 5′-GGAGAACCACCTTGTTGG-(N)20-GTTTAAGAGCTAAGCTGGAAAC-3′ were synthesized in a pooled format on microarray surfaces (GenScript Biotech, Inc.). Oligo pools were polymerase chain reaction (PCR)-amplified using Phusion Flash High-Fidelity PCR Master Mix (ThermoFisher Scientific) according to the manufacturer’s protocol with 1 ng/μl sgRNA template DNA, 1 μM forward primer (5′-GGAGAACCACCTTGTTGG-3′), and 1 μM reverse primer (5′- GTTTCCAGCTTAGCTCTTAAAC-3′) in 50 µl of total volume. The following cycle numbers were used: 1× (98°C for 3 min), 16× (98°C for 1 s, 54°C for 15 s, and 72°C for 20 s), and 1× (72°C for 5 min). PCR products were purified using NucleoSpin columns (Macherey-Nagel). The library vector pMB1-10x was prepared by restriction digestion with AarI (Thermo Fisher) at 37°C overnight. The digestion reaction was run on a 1% agarose gel followed by excision of the digested band and purification via NucleoSpin columns (Macherey-Nagel). 100 ng digested pMB1-10x and 2.4 ng amplified sgRNA library insert were assembled using Gibson Assembly Master Mix (NEB) in a 20 μl of reaction for 30 min. The reaction was purified using *P*-30 buffer exchange columns (Bio-Rad) that were equilibrated 5× with H_2_O and the eluted volume was transformed into 20 µl of MegaX DH10β cells (Thermo Fisher) by electroporation. *Escherichia coli* were recovered and cultured overnight in 100 ml of LB medium with 100 μg/ml ampicillin. The plasmid library was extracted using Midiprep (Qiagen). In parallel, a fraction of the transformation reaction was plated and used to determine the total number of transformed clones. The coverage was determined to be 1643× clones per sgRNA ensuring even representation of all library sgRNA sequences and their narrow distribution (Supplementary Fig. S2A). The quality of the cloned sgRNA library was determined by NGS on an Illumina MiniSeq (See below). MAGeCK [26] was used for library alignment. Narrow distribution of sgRNA sequences was confirmed with read counts for 96% of sgRNA sequences falling within a single order of magnitude (Supplementary Fig. S2A).

### Lentivirus production

Lenti-X 293T cells (Takara) were seeded at 65 000 cells per cm^2^ in 10 ml of media (DMEM, 10% FBS, 1% pen/strep) in a 10 cm dish and incubated overnight at 37°C, 5% CO_2_. On the next day, 5 μg sgRNA library plasmid, 2 μg psPAX2 (Addgene #12260), 2 μg pMD2.G (Addgene #12259), and 36 μl of Turbofect (Thermo Fisher) were mixed into 1.8 ml of serum-free DMEM (Gibco), vortexed briefly, incubated for 20 min at RT, and added to the cells. At 48 and 72 h post-transfection, the supernatant was harvested, passed through 0.45 µm filters (Millipore), and 20× concentrated using LentiX Concentrator (Takara) according to the manufacturer’s instructions. Aliquots were stored at −20°C.

### Knockout efficiency assessment with flow cytometry

HEK293∆RAF1:ER cells were seeded in 12-well plates at 50 000 cells/ml. The cells were transduced with lentivirally packaged gRNA construct targeting CD46 at a low multiplicity of infection (MOI) = 0.2 and incubated at 37°C, 5% CO_2_ for 48 h. After incubation, cells were selected with 2 µg/ml puromycin for 3 days. After 7, 10, 14, 18, and 20 days of infection, the cells were stained with the anti-CD46 antibody (Miltenyi Biotec), and the knockout efficiency was determined via flow cytometry analysis of >10 000 cells on a BD LSRFortessa II flow cytometer.

CD46 was used as a surrogate marker for perturbation efficiency because it is a ubiquitously expressed, non-essential surface protein whose loss can be quantified sensitively by flow cytometry. Similar strategies have been used previously as technical readouts for perturbation efficiency in pooled CRISPR screens [27, 28].

### Direct capture perturb-seq CRISPR screens

**Figure 2.**
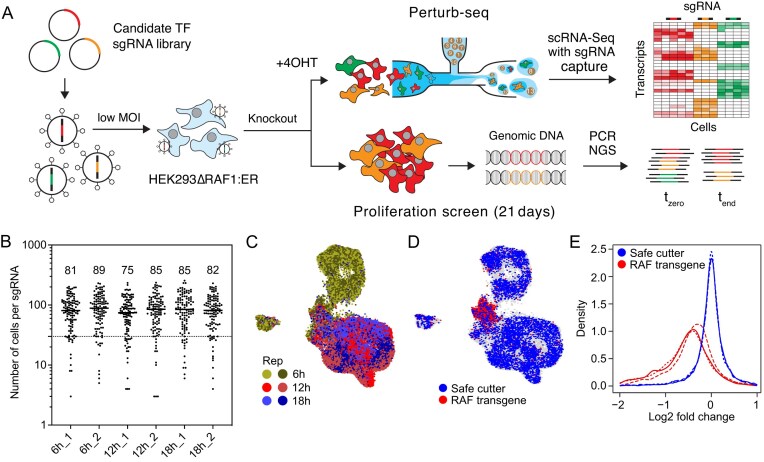
Perturb-seq and proliferation screens in HEK293∆RAF1:ER. (**A**) Schematic of Perturb-seq screens (top panel) and proliferation CRISPR screens (bottom panel). (**B**) Median number and distribution of cells per sgRNA detected in the respective Perturb-seq samples. The dashed line indicates the cut-off at 30 cells per sgRNA. (**C**) UMAP integration of Perturb-seq samples from three different time points with two replicates each. (**D**) Distribution of the safe cutter sgRNA cells and the RAF1-knockout cells on the UMAP clusters. (**E**) Histograms of the log_2_ fold change of previously identified RAF/MAPK target genes (Fig. 1) between RAF1-knockout (red) and the safe cutter control cells (blue), relative to the non-target control cells. Dashed line: 6 h time point, Dotted line: 12 h time point, Solid Line: 18 h time point. Perturb-seq screens were performed in duplicate.

HEK293∆RAF1:ER cells were transduced with lentivirally packaged sgRNA library at a MOI = 0.2 and 1000× coverage in two replicates. The low MOI and high coverage were used to reduce the frequency of multiple-infected cells thus only one gene was knocked out in each cell and ensure even distribution of the sgRNA library. This resulted in the detection of one sgRNA in the majority of captured cells (Supplementary Fig. S2B). The median number of analyzable cells per sgRNA varied between experiments, ranging from 75 to 89 cells (Fig. 2B). Cells were then cultured in DMEM low glucose without phenol red with 10% FBS (Pan Biotech) and 1% pen/strep (Sigma–Aldrich) in a 37°C incubator with 5% CO_2_. Forty-eight hours after transduction, transduced cells were selected with puromycin (2 μg/ml) for 96 h. After selection, the top 20% mCherry positive cells were sorted 8 days post infection using a BD FACSAria II flow cytometer to increase sgRNA capture efficiency by the 10x Genomics Gel Beads. A total of 3 million cells were sorted. For the Perturb-seq screen, 500 000 of the sorted cells were reseeded in full medium in three wells of a 12-well plate and incubated at 37°C, 5% CO_2_. At day 10 post infection, the sorted cells were stimulated with 0.5 µM 4OHT (Sigma–Aldrich, H7904) for 6, 12, and 18 h. After the incubation time, the cells were harvested followed by single-cell RNA sequencing (scRNA-seq) following the 10x Genomics Chromium Next GEM Single Cell 3′ Reagent Kits v3.1 (Dual Index) with Feature Barcode technology for CRISPR Screening protocol.

### Pooled proliferation CRISPR screen

The remaining 2.5 million sorted HEK293∆RAF1:ER cells from day 8 post infection were reseeded in full medium in a six-well plate and incubated at 37°C, 5% CO_2_. On day 18 post infection, 0.5 µM 4OHT was added for 48 h to induce the RAF1 transgene expression and apoptosis of the induced cells. After 48 h, the dead cells were detached and removed with the medium. The living cells were reseeded in tamoxifen containing medium and incubated at 37°C, 5% CO_2_ for 48 h more to increase the cell selection efficiency. Aliquots of 500 000 cells from the 48 h induction time point were taken. The cells were centrifuged, and the cell pellets were frozen down for later analysis via NGS.

### Genomic DNA extraction and PCR recovery of gRNA sequences

The genomic DNA (gDNA) was extracted from the HEK293∆RAF1:ER cells using the Qiagen Genomic DNA extraction kit according to the manufacturer’s instructions. Two nested PCR reactions were performed to amplify the sgRNA cassette from the extracted gDNA. For the first PCR reactions, 5 μg gDNA, 0.3 μM forward (5′-GGCTTGGATTTCTATAACTTCGTATAGCA-3′) and reverse (5′-CGGGGACTGTGGGCGATGTG-3′) primer, 200 μM dNTP mix, 1× Titanium Taq buffer, and 2 μl Titanium Taq polymerase (Takara) were mixed in 50 µl total volume. The PCR reaction was run using the following cycles: 1× (94°C, 3 min), 20× (94°C, 30 s, 65°C, 10 s, 72°C, 20 s), and 1× (68°C, 2 min). For the second PCR reactions, 5 μl first-round PCR, 0.5 μM forward (5′-AATGATACGGCGACCACCGAGATCTACACACACTCT TTCCCTACACGACGCTCTTCCGATCTTCCCTTG GAGAACCACCTTGTTGG-3′) and reverse (5′-CAAGCAGAAGACGGCATACGAGAT-(N)_6_-GTGACTGGAGTTCAGACGTGTGCTCTTCCGATC-3′) primer where (N)_6_ is a 6 nt index for sequencing on the Illumina MiniSeq platform, 200 μM dNTP mix, 1× Titanium Taq buffer and 2 μl Titanium Taq (Takara). PCR cycles were: 1× (94°C, 3 min), 20× (94°C, 30 s, 55°C, 10 s, 72°C, 20 s), and 1× (68°C, 2 min). The PCR product (325 bp) was purified from a 1% agarose gel via NucleoSpin columns (Macherey-Nagel). NGS was performed on an Illumina MiniSeq using a MiniSeq Mid Output Kit (300-cycles) using paired-end 150 strategy according to the manufacturer’s instructions.

### Proliferation screen data analysis

The proliferation screen data analysis was performed using MAGeCK [26]. In short, sgRNA read count files were computed from the raw CRISPR fastq files using the count function. The MAGeCK MLE command was then used to calculate the MAGeCK Beta score, Wald-*P* values, and false discovery rates for enrichment and depletion of each guide at day 20 and day 22 after tamoxifen induction compared to the plasmid library. Wald-*P* values were adjusted using Bonferroni correction method in R (Supplementary Table S2).

### scRNA-seq screen data analysis

Cell Ranger (10x Genomics) Version 6.1.1 was used for scRNA-seq data processing (https://www.10xgenomics.com/support/software/cell-ranger/latest/analysis/running-pipelines/cr-gex-co). Sequencing reads coming from the gene expression library were mapped to the GRCh38-1.2.0 genome reference compiled by 10x Genomics for Cell Ranger. Guide RNA reads were mapped simultaneously to a sgRNA feature reference. The combination of standard and targeted RNA-seq was processed by pooling the fastq files and subsequent Cell Ranger analysis, thereby avoiding duplicate counts for the same molecules as reads with the same UMIs are collapsed. Count matrices were then used as input into the Seurat R package [29] to perform downstream analyses. Differential expression was called based on pseudo bulks using the R-library glmGamPoi version 1.10.2 [30]. Cells with >1 or 0 detectable sgRNA and sgRNAs with <30 analyzable cells were omitted from further analysis, resulting in approximately 10 000 analyzable cells per sample expressing exactly one sgRNA and a median sgRNA UMI count of 26–84 (Fig. 2B and Supplementary Fig. S2C).

**Figure 3.**
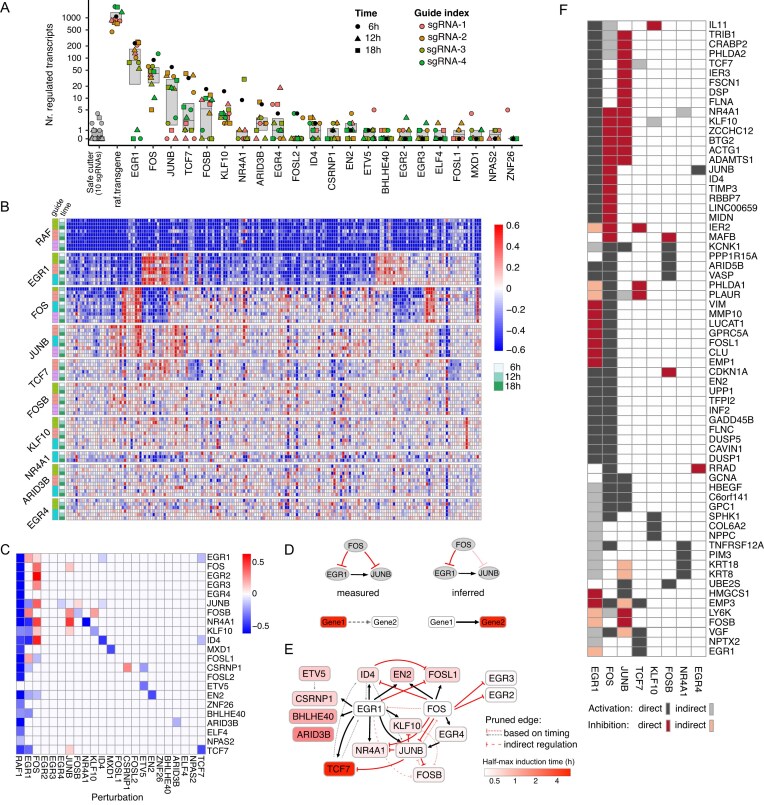
Summary of Perturb-seq screen results and model of transcriptional interactions between candidate TFs. (**A**) Number of significantly deregulated transcripts (adjusted *P*-value < 0.05) following perturbation of the indicated candidate TF separated by sgRNAs and time points. (**B**) Heatmap of the log_2_ fold change of the 140 genes included in the modified TAP-seq for the perturbations with the strongest transcriptional response. The results from perturbed target genes separated by sgRNAs and time points are shown. Negative values indicate lower and positive values higher target transcript levels in the perturbed cells relative to cells expressing non-target control sgRNAs. (**C**) Core heatmap of candidate TF expression changes following the perturbation of all 22 TFs and RAF1, showing the log_2_ fold change upon perturbation for the significantly differentially expressed TFs (adjusted *P*-value < 0.05). (**D**) Example for the removal of edges from coherent feed forward loops: The measured perturbation data are compatible with a feed-forward loop, where FOS inhibits JUNB directly and indirectly, but it is also compatible with a cascade, where FOS inhibits JUNB via EGR1 only. To generate the most parsimonious network, we removed the feed forward loop from FOS to JUNB in the inferred network and termed it “indirect.” Edges from late (red) to early (white) induced genes are dotted lines while the opposite orientation is represented by solid lines. (**E**) *De novo* model of the TF core network showing directional transcriptional interactions between all perturbed TFs with inferred interaction type: Black = activating, Red = inhibiting. Transparent edges indicate the removed feed forward loops. Transcription factors are color-coded by their half-maximal induction time. (**F**) Summary of target genes that are directly or indirectly co-regulated by more than one candidate TF.

Integration of UMAPs from different cell-cycle phases showed that cells within the major clusters were represented across G1, S, and G2/M phases, suggesting that cell-cycle heterogeneity was not the dominant source of the observed variation (Supplementary Fig. S2D). Pseudo-bulk analysis of the previously identified RAF–MAPK targets (Fig 1B) showed a normal distribution centered around zero in safe cutter controls, whereas RAF1 knockouts displayed a clear shift toward negative log2 fold changes (Fig. 2E). These findings demonstrate that the transcriptional changes observed in control cells are specifically driven by RAF1 activation and not by Cas9-induced double-strand breaks. Examination of individual RAF1 sgRNAs confirmed that three guides were effective, with 12%–37% of RAF1-induced genes (identified by bulk RNA-seq) significantly deregulated (Supplementary Fig. S2E). The fourth sgRNA was represented in fewer than 30 cells and was excluded from analysis. Because target gene induction was similar across time points, sgRNAs and time points were combined to define overall fold changes and applied Fisher’s method to combine sgRNAs and time points resulting in a “combined *P*-value” to identify significantly deregulated targets, resulting in a core heatmap of candidate TFs (Fig. 3C).

### Network inference

An initial core network was constructed as follows: (i) We added edges connecting perturbed TFs that upon perturbation had significant influence on the expression of another TF in the network. To do so, we selected the “combined” *P*-values (Fisher’s method, see above), performed multiple testing correction using the Benjamini–Hochberg method, and retained edges with adjusted *P*-values below 0.05. Edges were signed based on the negative sign of the fold-changes upon KO. (ii) We filtered these edges based on consistency in timing, i.e. those interactions where the regulator had earlier induction than the target when RAF signaling was induced. For this step we used the time-series transcriptome data (Figure 1), and for each gene defined a half-maximal induction by selecting the time points before and after half-maximal induction, and linear interpolation. (iii) We resolved feed-forward loops (A → B → C and A →C) by removing the direct link A → C.

### Gene expression library amplification (TAP-seq)

TAP-seq was performed by using PCR amplification directly from pre-amplified complementary DNA (cDNA) libraries, increasing sensitivity without loss of specificity (Supplementary Fig. S4A). This modification reduces contamination risk, allows retrospective amplification of new gene panels, and was applied here to 140 RAF1-responsive genes (Supplementary Fig. S4B). For the generation of suitable primers, we used the BAM file of an untargeted 10× run (6 h 4OHT) in the TAP-seq R package (https://github.com/argschwind/TAPseq) and workflow that is delineated in the package vignette [17]. Deviations from the TAP-seq workflow were as follows: (i) We only used the inner primer generation procedure, i.e. at 150–300 bp from inferred poly(A) sites. (ii) Originally primers for one major poly(A) site per gene were generated, instead we generated primers for all top poly(A) sites amounting to >70% of the total poly(A) score (i.e. coverage) per gene. (iii) Additional filter steps on poly(A) level, i.e. removal of minor poly(A) sites in adjacent poly(A) signals within 100 bp, and on primer level, i.e. removal of redundant primers within 500 bp of poly(A) signal and manual filter of badly designed primer e.g. in non-expressed regions. The final primer list is provided in the Supplementary Table S3. Targeted primers were ordered from IDT in desalted format with 5′-GTGACTGGAGTTCAGACGTGTGCTCTTCCGATCT-3′ as PCR handle at 5′ ends. 100 µl PCR was performed as follows: 100 ng amplified cDNA (Step 2.3 of 10× Genomics 3′scRNA), 2.5 µl of 100 µM Pooled targeted Primer Mix, 4 µl of 10 µM partial Read 1 (sequence from 10× Genomics manual, ordered as normal (desalted) primer from IDT 5′-CTACACGACGCTCTTCCGATCT-3′, 50 µL KAPA HiFi HS RM (Roche, KK2601). The PCR was performed with the following cycle numbers: 1× (95°C, 3 min), 10× (98°C, 20 s, 67°C, 60 s, 72°C, 60 s), and 1× (72°C, 5 min). The PCR product was cleaned using SPRIselect beads (Beckman Coulter) as a double sided size selection (as described in the Tips & Best Practices Section of a typical 10× Genomics procedure) with 0.6× as first SPRI and 1.2× as second SPRIselect steps. For adding indices, maximally 10 ng of the first cleaned PCR product were then mixed with 20 µl dual Index TT Set A from 10× genomics, 50 µl KAPA HiFi HS RM (Roche, KK2601) in a 100 µl reaction. The PCR was performed with the following cycle numbers: 1× (95°C, 3 min), 1× (98°C, 45 s), 10× (98°C, 20 s, 54°C, 30 s, 72°C, 20 s), and 1× (72°C, 1 min). As the amplicons are bigger now, we changed the SPRI concentration to 0.55× as first SPRI and 1.2× as second SPRIselect steps. Library quality was assessed using the TAPE station. The approach maintained specificity in safe cutter controls while substantially increasing the proportion of significantly deregulated genes in RAF1 knockouts and candidate TF perturbations, including EGR1 and FOS (Supplementary Fig. S4C). Therefore, targeted amplification increased sensitivity for biologically relevant RAF1-responsive transcripts and improved the ability to resolve regulator-specific effects in the Perturb-seq screen.

### EGR1 and FOS knockout clonal line production

HEK293∆RAF1:ER single knockout clones for FOS and EGR1 genes were generated using CRISPR–Cas9. Two sgRNAs per gene were designed to target 500 bp sequences surrounding the 5′ end using CRISPOR [31]. The sgRNAs and their reverse complements were synthesized and cloned into the px459 vector [32]. For the EGR1 gene knockout, the following oligo sequences were used: 5′-CACCGGGCCATGTACGTCACGACGG-3′ and 5′-CACCGGGACAACTACCCTAAGCTGG-3′ targeting the promoter and the exon regions respectively. For the FOS gene knockout, the following oligo sequence was used to target the promoter region; 5′-CACCGGATTAGGACACGCGCCAAGG-3′ and the exon region, 5′-CACCGGAGAGAGGCTATCCCCGGCCG-3′. The oligo for FOS exon contains an added G at the 5′ end of the gRNA to facilitate U6 promoter mediated transcription. Cells were transfected with these vectors following the Lipofectamine 2000 (ThermoFisher Scientific) and selected with puromycin (250 ng/ml) for 36 h starting 24 h after transfection. Following selection, we performed clonal dilution. Cells were seeded in 96-well plates and wells with individual clones were screened via PCR to identify successful 5′ end deletions. Positive hits were further validated at RNA and protein levels using qPCR and Western blot, to confirm the absence of FOS and EGR1 gene expression.

Starting from single knockout clones of EGR1 and FOS, double knockout clones were generated using Alt-R™ S.p. Cas9 Nuclease V3 from IDT used with guides designed with the manufacturer’s design tool (https://eu.idtdna.com/site/order/designtool/index/CRISPR_CUSTOM) (Supplementary Table S4). Transfection was performed following the Lipofectamine CRISPRMAX (ThermoFisher Scientific), using less RNA amounts depending on the number of sgRNAs used per transfection. Two days after transfection, clonal dilution was performed. Isolation of gDNA was done with Quick-DNA-96 Kits (Zymo), for PCRs KAPA HiFi HS RM (Roche) and different primer sets spanning regions out and/or inside expected deletions were used. PCR product size was analyzed with TAPE Station from Agilent. Clones with successful deletions based on the PCR result were then analyzed using western blots with antibodies against FOS or EGR1 and pERK antibody (Cell Signaling Technology) to identify clones that lack expression of the respective TFs and still induce MAPK signaling when the RAF1–CR3 kinase domain is activated with 4OHT. KO clones were cultivated in low glucose DMEM without Phenol red (Sigma–Aldrich) supplemented with stable Glutamine (PAN-Biotech) and FBS (PAN-Biotech).

### Bulk RNA-seq

For bulk RNA sequencing of single and combinatorial EGR1 and FOS knockout clonal lines cells were treated with 0.5 µM 4OHT for 6, 12, and 18 h. To account for differences in cell density, two solvent control wells were collected each at first and last lysing time of the treatments. RNA isolation was done with RNeasy Kits from Qiagen without any DNA elimination. RNA concentration was measured with the Implen nanophotometer and 250–450 ng per sample were used for Library preparation with QuantSeq 3′ mRNA-Seq V2 (Lexogen). For Index PCR, 18 cycles were used according to the manual. Upon quantification with Universal KAPA Library Quantification Kit for Illumina (Roche) Libraries were pooled and sent for sequencing.

For bulk RNA sequencing of TCF7 knockout cells after MAPK and/or Wnt signaling activation, TCF7 and Safe-cutter knockout cells were made by transducing the cells with lentiviruses made from pMB1-sgTCF7-2 and pMB1-sgSafe-cutter-1 vectors without the 10× Genomics capture sequence 1 in the stem-loop of the Cas9-tracr sequence. The cells were selected with 2 µg/ml puromycin and incubated for 10 days. After the incubation period, the cells were treated with 0.5 µM 4OHT for 12 h, 10 µM CHIR99021 for 6 h, or the combination of both treatments. The cells were then harvested and the total RNA was extracted using the RNeasy Mini Kit (Qiagen, 74106), according to the manufacturer’s instructions. The RNA was then quantified using a NanoDrop One, libraries were prepared using Watchmaker RNA Library Prep with PolarisT Depletion according to manufacturer’s instruction, and subsequently sequenced on an Illumina NovaSeq X. Data were processed using the snakemake pipeline rna-seq-star-deseq2 and subsequent analysis using DeSeq2 in R.

### Quantitative real-time polymerase chain reaction (qRT-PCR)

HEK293∆RAF1:ER EGR1, TCF7, and Safe-cutter knockout cells were generated as before. For the MAPK and Wnt signaling pathway activation the cells were treated with 0.5 µM 4OHT for 12 h and 10 µM CHIR99021 for 24 h. For the EGR1–TCF7 transcriptional positive feedback loop, the cells were then treated with 0.5 µM 4OHT for 1 h while the cells for checking the TCF7–EGR1 positive feedback loop were treated with 0.5 µM 4OHT for 12 h.

Total RNA was isolated for qRT-PCR with a RNeasy Mini Kit (Qiagen, 74106) according to the manufacturer’s instructions, and quantified using a Nanodrop One. cDNA was prepared from total RNA using a RevertAid H Minus First Strand cDNA Synthesis Kit (ThermoFischer Scientific, K1632). Quantitative PCR was performed using ORA qPCR Green ROX L Mix (highQu, QPD0105) using a LightCycler^®^ 480 System (Roche). Analysis was performed using the 2^-∆∆Ct^ method. GAPDH was used as a housekeeping gene. The following primers were used: GAPDH_for: CTGGTAAAGTGGATATTGTTGCCAT, GAPDH_rev: TGGAATCATATTGGAACATGTAAACC, EGR1_for: CTTCAACCCTCAGGCGGACA, EGR1_rev: GGAAAAGCGGCCAGTATAGGT, TCF7_for: CTGACCTCTCTGGCTTCTACTC, TCF7_rev: CAGAACCTAGCATCAAGGATGGG, AXIN2_for: CAAACTTTCGCCAACCGTGGTTG, AXIN2_rev: GGTGCAAAGACATAGCCAGAACC.

### Processing of bulk, multiplexed QuantSeq data

bcl2fastq (v2.20.0 by Illumina) was used to demultiplex and convert raw sequencing data to fastq files. We designed a Snakemake (v7.18.2) workflow in which BBMap’s BBDuk (v39.01) was used to trim adapters, STAR (v2.7.10b) to align reads to the GENCODE GRCh38.p13 (v39) geneset, umitools (v1.1.4) to extract and deduplicate UMIs, and subread’s featureCounts (v2.0.6) to count mapped reads on gene level.

## Results

### Identification of transcription factors up-regulated by RAF1-induction

To identify transcripts that are up-regulated by RAF–MAPK activation, we used a previously established HEK293 cell line, termed HEK293ΔRAF1:ER, in which a tamoxifen-inducible RAF1–CR3 kinase domain was introduced [21]. Consequently, RAF1 activity can be precisely regulated, allowing the identification of RAF1–MAPK response genes (Fig. 1A). In contrast to cell culture systems stimulated by growth factors, this model of RAF–MAPK signaling is activated independently of the upstream G-protein RAS, thus minimizing pathway divergence and feedback mechanisms. We induced RAF activity with 4OHT over periods of 0.5–8 h and monitored the changes in the transcriptome of the cells via bulk RNA-sequencing (RNA-seq). Over the full time series, we detected a total of 1142 significantly up-regulated genes (Fig. 1B). From this dataset, 22 transcription factors (TFs) that were up-regulated at different time points after RAF1 induction were selected for further analysis. The basal expression levels of identified candidate TFs varied over several orders of magnitude and their level of up-regulation upon RAF1 induction was independent of their basal expression level (Fig. 1C).

Pulsed induction of RAF1 (Fig. 1D) further allowed the categorization of the candidate TFs into three distinct response classes. The first class comprises classical immediate early genes that are rapidly induced upon signal induction and whose mRNAs are rather short lived, resulting in rapid decay after the pulses ended. Examples of these transcripts are the rapidly induced EGR gene family members, FOS, FOSB, and JUNB, which increased to maximum mRNA levels directly after induction and decreased quickly after 4OHT removal. The second class comprises rather rapidly induced, long-lived transcripts that are induced within the first 1–2 h and remain high even after the pulse has ended, termed immediate late genes. These include, for instance, the transcripts of FOSL1 and FOSL2. A third class of transcription factors is only induced with delay. For several of those transcripts, the response is limited to long pulses of induction. For instance, TCF7 is only induced after 4 h and reaches a maximum response after 8 h of induction. We furthermore calculated the time when the transcripts reach half-maximal expression by interpolating between the measured time points. We found that the selected transcripts cover half-maximal induction times between 20 min and 5 h (Table 3).

**Table 3.** Selected candidate genes and their classification based on their response time to RAF1 induction [9]. IEG = immediate-early genes, ILG = immediate-late genes, DEG = delayed-early genes. SRG = secondary response gene

Gene Symbol	Name	Class [9]	Half-maximal induction (h:min)
**EGR1**	Early Growth Response 1	IEG	0:17
**FOS**	Fos Proto-Oncogene, AP-1 Transcription Factor Subunit	IEG	0:19
**EGR2**	Early Growth Response 2	IEG	0:22
**EGR3**	Early Growth Response 3	IEG	0:25
**EGR4**	Early Growth Response 4	ILG	0:29
**JUNB**	JunB Proto-Oncogene	IEG	0:33
**FOSB**	FosB Proto-Oncogene	IEG	0:37
**NR4A1**	Nuclear Receptor Subfamily 4 Group A Member 1	ILG	0:47
**KLF10**	KLF Transcription Factor 10	DEG	0:49
**ID4**	Inhibitor Of DNA Binding 4	DEG	1:05
**MXD1**	MAX Dimerization Protein 1	DEG	1:13
**FOSL1**	FOS Like 1	ILG	1:17
**CSRNP1**	Cysteine And Serine Rich Nuclear Protein 1	SRG	1:19
**FOSL2**	FOS Like 2	ILG	1:23
**ETV5**	ETS Variant Transcription Factor 5	IEG	1:31
**EN2**	Engrailed Homeobox 2	SRG	1:37
**ZNF26**	Zinc Finger Protein 26	DEG	2:09
**BHLHE40**	Basic Helix-Loop-Helix Family Member E40	DEG	2:15
**ARID3B**	AT-Rich Interaction Domain 3B	SRG	3:03
**ELF4**	E74 Like ETS Transcription Factor 4	SRG	3:10
**NPAS2**	Neuronal PAS Domain Protein 2	SRG	3:16
**TCF7**	Transcription Factor 7	SRG	5:08

The selected transcription factors exhibit distinct kinetics in the RAF1-induced transcriptional response, encompassing transcripts previously classified as immediate-early (IEG), immediate-late (ILG), delayed-early (DEG), and secondary response genes (SRG) [9]. While these induction dynamics suggest whether a factor acts early or late in the RAF response, they do not reveal whether, or how, the induced TFs functionally contribute to transcriptional network regulation downstream of RAF–MAPK. Together, these results show that RAF1 activation triggers a temporally ordered transcription factor cascade composed of rapid immediate-early responders and delayed secondary regulators, providing a framework for downstream network architecture. To systematically define the transcripts controlled by the 22 TFs listed in Table 3, we employed Perturb-seq [15, 16].

### Perturb-seq reveals the transcriptional targets of RAF1-induced TFs

To identify the transcriptional targets of the selected 22 candidate TFs, we performed pooled CRISPR/Cas9 screens with single cell RNA-seq read-out, via direct-capture Perturb-seq [19], as well as pooled CRISPR screens with proliferation read-out (Fig. 2A). For that purpose, we designed and cloned a pooled sgRNA library, targeting the 22 candidate TFs. In addition, the library contained 4 sgRNAs against the 4OHT-inducible RAF1 transgene as a positive control. The sgRNA library was then transduced into HEK293∆RAF1:ER cells at low multiplicity of infection (MOI = 0.2). Following a 10-day period to allow for CRISPR/Cas9 gene editing (Supplementary Fig. S1), RAF1 was induced by 4OHT for 6, 12, and 18 h, respectively, to capture time-resolved transcription profile changes from each of the 22 perturbed candidate TFs. Cells were then analyzed via scRNA-seq using “Feature Barcode technology” (10X Genomics).

Perturb-seq data passed stringent QC, with expected guide representation, reproducible time-dependent transcriptional separation, and appropriate performance of positive and negative controls (Fig. 2B–E and Supplementary Fig. S2). More specifically, integration of UMAPs from Perturb-seq experiments at 6, 12, and 18 h after RAF1 induction revealed that cells from the 12- and 18-h time points clustered separately from those at 6 h (Fig. 2C). This indicates that major transcriptional changes occur between 6 and 12 h, highlighting the temporal dynamics of the RAF1 response. To validate the system, we compared RAF1-transgene knockout cells to safe cutter control cells. RAF1 knockouts formed a distinct cluster, clearly separated from the controls (Fig. 2D), confirming the absence of a transcriptional response to RAF1 induction in these cells.

These data established a robust framework for defining functional regulators of the RAF1 transcriptional response. Thus, the screen quality controls demonstrate that the observed transcriptional phenotypes reflect specific perturbation of the RAF1-responsive network rather than technical or cell-cycle-driven variation. Taken together, these results demonstrate the high technical quality of the generated Perturb-seq data, which formed the basis for further analyses.

### Proliferation CRISPR screens after candidate TF perturbation

We next asked whether knockout of individual transcription factors affects cell growth, using a CRISPR/Cas9 proliferation screen performed in HEK293∆RAF1:ER cells with and without RAF induction (Fig. 2A and Supplementary Fig. S3). Most TF knockouts showed no significant depletion or enrichment compared to non-targeting controls. Guide RNAs targeting FOS and EGR2 were significantly depleted under both conditions, consistent with their known roles in cell fitness [33, 34]. In addition, knockouts of ELF4, FOSL1, FOSB, and EGR3 also showed significant depletion, though to a lesser extent. By contrast, knockout of the ∆RAF1:ER transgene was strongly enriched, but only upon RAF activation, in line with previous reports that prolonged RAF–MAPK activation in HEK293∆RAF1:ER cells induces apoptosis [20]. Overall, this screen demonstrates that TF knockouts do not cause major growth disadvantages, ensuring sufficient guide representation for subsequent Perturb-seq experiments (Supplementary Fig. S3).

### Number of deregulated target genes varies greatly between TFs

Perturb-seq screens are powerful but limited by sequencing costs, low sensitivity for weakly expressed genes, and small effect sizes. To address this, we adapted Targeted Perturb-seq (TAP-seq, [17]). We then combined the targeted and untargeted Perturb-seq data to optimally analyze the 140 selected transcripts at high sequencing depth. In addition, it covers the entire transcriptome from the standard gene expression library. Figure 3 summarizes the results of a pseudo-bulk analysis of the combined targeted and untargeted Perturb-seq screens at 6, 12, and 18 h after RAF1 induction, analyzed separately for each sgRNA. Consistent with Supplementary Fig. S4C, negative control sgRNAs produced no significant deregulation, whereas RAF1 knockouts affected 500–1000 genes across all time points (Fig. 3A). The effects of TF perturbations varied widely: EGR1 knockouts deregulated hundreds of targets, while more than half of the investigated TFs had little or no effect. Heatmap analysis (Fig. 3B) confirmed that RAF1 loss blocked induction of its targets, with most changes evident by 6–12 h and minimal additional effects at 18 h, consistent with known RAF1 induction kinetics [2]. These results reveal that the RAF1-induced transcriptional cascade is hierarchical, with EGR1 and a small number of additional TFs exerting disproportionately large control over downstream gene expression.

### Perturb-seq results are highly reproducible

To technically validate the Perturb-seq findings, we analyzed EGR1 and FOS single and double knockout clones in HEK293∆RAF1:ER cells by bulk RNA-seq. The results closely resembled the Perturb-seq screen results, showing consistent directionality of regulation and strong correlations for the 140 TAP-seq transcripts (*r* = 0.69–0.8; Supplementary Fig. S5A and B). The stronger effects observed in the clonal bulk RNA-seq knockout lines likely reflect the combination of complete gene deletion and the homogeneous perturbation across the population relative to pooled single-cell Perturb-seq, in which variable editing efficiency can attenuate apparent effect sizes. Combinatorial EGR1–FOS knockouts clustered with the single knockout of FOS across both methods, suggesting that FOS is epistatic to EGR1 in the regulation of the RAF–MAPK response (Supplementary Fig. S5C). Although the global profile of the double knockout resembled FOS loss, it also uncovered a synergistic role of EGR1 and FOS in up-regulating EGR2, EGR3, and EGR4, which were strongly induced only in the double knockout (Supplementary Fig. S5D). This suggests that the RAF–MAPK transcriptional program is not simply additive, but contains both dominant and synergistic regulatory relationships, allowing selective expression of specific downstream genes such as the EGR family.

### De-novo construction of a TF core network identifies a transcriptional positive feedback loop between EGR1 and TCF7

A core heatmap of candidate TFs was constructed to analyse how the perturbation of each TF influences the transcriptional profile of the other candidate TFs (Fig. 3C). Most TF knockouts also reduced their own transcript levels, consistent with nonsense-mediated decay of Cas9-induced stop-gain mutations [35]. In contrast, EGR1, FOS, and CSRNP1 were up-regulated, likely reflecting auto-regulatory feedback in which loss of protein function enhances transcription of mutant mRNA. To further assess these self-target effects at the level of individual sgRNAs, we quantified the log_2_ fold change of each targeted transcript after perturbation with its corresponding sgRNA across the 6, 12, and 18 h time points (Supplementary Fig. S6). This analysis showed that target transcript changes were generally consistent across multiple sgRNAs per gene, while also revealing sgRNA-level variability in the magnitude and significance of the effect. Within the core network, EGR1 emerged as a central activator and FOS as a central inhibitor, often acting in opposition on shared targets such as JUNB, KLF10, ID4, and NR4A1. FOSL1 was oppositely co-regulated, while EN2 was co-activated by both TFs. These findings highlight EGR1 and FOS as orthogonal regulators, orchestrating the RAF1-driven transcriptional response.

To assess biological plausibility, we integrated time-series RNA-seq data (Fig. 1), removing nine edges where target activation preceded upstream TF induction, most likely representing indirect inhibitory effects. The final network also included a putative feed-forward loop (EGR1 → JUNB → FOSB and EGR1 → FOSB), which could not be distinguished from a simpler model based on perturbation data alone. Applying parsimony, we excluded such ambiguous loops, yielding a refined core network (Fig. 3E). To contextualize these inferred TF–TF interactions, we compared the refined core network with regulatory interactions annotated in OmniPath/DoRothEA (Supplementary Fig. S7). Of the 23 inferred non-pruned interactions in the core network, 16 were represented in OmniPath/DoRothEA, whereas seven were not found in these prior-knowledge resources. Among the annotated interactions, nine were supported by published experimental evidence and seven were listed as inferred interactions without associated experimental support. We then extended the analysis to target genes beyond the 22 candidate TFs. Figure 3F presents target genes co-regulated by the candidate TFs with the broadest regulatory impact, including EGR1, FOS, JUNB, TCF7, KLF10, FOSB, NR4A1, and EGR4. Together, the inferred core network identifies EGR1 and FOS as the principal opposing hubs of the RAF-MAPK transcriptional response, where EGR1 interacts with TCF7 in a positive feedback loop.

### EGR1–TCF7 positive feedback loop links RAF–MAPK and Wnt transcriptional programs

**Figure 4.**
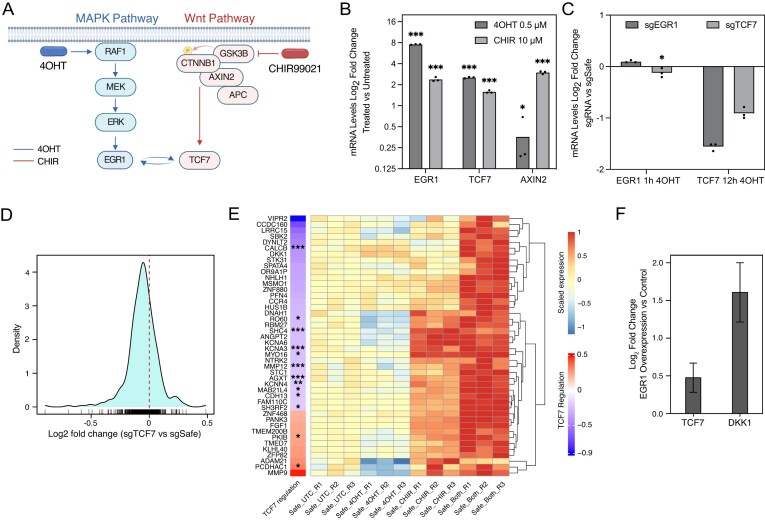
Characterization of MAPK and Wnt signaling pathways cross-talk through TCF7. (**A**) Simplified schematic of the MAPK- and Wnt-signaling pathways, including the transcriptional EGR1–TCF7 positive feedback loop. RAF–MEK–ERK pathway is activated by 4OHT treatment. Wnt signaling is activated by the GSK3B inhibitor CHIR99021. Created in BioRender. Böttcher, M. (2026) https://BioRender.com/8k6fcao. (**B**) RT qPCR measurement of EGR1, TCF7, and AXIN2 mRNA levels after treatment of HEK293∆RAF1:ER cells with 0.5 µM 4OHT or 10 µM CHIR99021. Values represent the mean of biological replicates (*n* = 3). (**C**) Validation of the transcriptional EGR1–TCF7 positive feedback loop identified in the Perturb-seq screens through RT qPCR measurement of EGR1 and TCF7 mRNA level fold change after perturbation of EGR1 and TCF7. Values represent the mean of biological replicates (*n* = 3). (**D**) Density plot showing the log_2_ fold-change distribution of all 364 synergistically activated Wnt signaling-associated genes in TCF7 knockout cells compared with safe cutter control cells under combined 4OHT and CHIR99021 treatment. (**E**) Heatmap of transcriptional changes detected by bulk RNA-seq in cells treated with 0.5 µM 4OHT for 12 h, 10 µM CHIR99021 for 6 h, or the combination of both treatments. Shown are synergistically activated Wnt signaling-associated genes that also display TCF7-dependent regulation under combined 4OHT and CHIR99021 treatment, as determined by comparing TCF7-targeting sgRNAs with safe cutter control sgRNAs. (**F**) Reanalysis of a RNA-sequencing dataset (GSE209899) showing DKK1 and TCF7 mRNA log_2_ fold changes after overexpression of EGR1 in Hela cells. Values represent the mean of biological replicates (*n* = 3). * Adj. *P*-value < 0.05, ** Adj. *P*-value < 0.01, *** Adj. *P*-value < 0.001.

Among all nodes in the core network (Fig. 3E), only one transcriptional positive feedback loop was identified—between EGR1 and TCF7. EGR1 is a well-established downstream effector of MAPK signaling induced by 4OHT in HEK293∆RAF1:ER cells, whereas TCF7, also induced by 4OHT (Fig. 1), is best known as a transcription factor in the Wnt pathway. This suggested that TCF7 might function as a transcriptional link between MAPK- and Wnt-driven transcriptional programs through its transcriptional relationship with EGR1. To test this, we activated Wnt signaling in HEK293 cells with the GSK3β inhibitor CHIR99021, which stabilizes β-catenin and activates canonical Wnt signaling (Fig. 4A). Expression analysis confirmed pathway specificity: 4OHT strongly induced EGR1 (188-fold) and TCF7 (5.8-fold) but only marginally affected the Wnt marker AXIN2 (1.3-fold), indicating no activation of Wnt signaling (Fig. 4B). In contrast, CHIR99021 robustly up-regulated AXIN2 (8-fold) and TCF7 (3-fold), consistent with Wnt activation, and also increased EGR1 expression (5.2-fold). We next validated the feedback loop by measuring EGR1 and TCF7 after their respective knockouts. TCF7 loss modestly but significantly reduced EGR1 expression, while EGR1 knockout lowered TCF7 levels (∼0.7-fold), confirming mutual activation (Fig. 4C).

To assess whether publicly available chromatin-binding data support this inferred regulatory relationship, we examined ENCODE ChIP-seq datasets for EGR1 and TCF7 at the EGR1 and TCF7 loci and visualized these tracks together with full-length RNA-seq coverage from control and 4OHT-treated RAF1:ER cells (Supplementary Fig. S8). At the EGR1 locus, ENCODE ChIP-seq tracks showed promoter-proximal EGR1 occupancy, consistent with potential autoregulation, whereas TCF7 binding was not evident in the displayed datasets. At the TCF7 locus, promoter-proximal binding was observed for both EGR1 and TCF7, providing external support for a potential direct regulatory connection between EGR1 and TCF7. Full-length RNA-seq coverage confirmed expression of the corresponding transcripts in 4OHT-treated RAF1:ER cells. Thus, the inferred EGR1–TCF7 feedback loop may constitute a transcriptional bridge through which RAF–MAPK signaling can potentiate Wnt-responsive gene expression, although direct chromatin engagement and promoter-level regulation in the RAF1:ER system remain to be experimentally validated.

### TCF7 contributes to the transcriptional integration of MAPK and Wnt pathway outputs

To further test whether TCF7 contributes to the transcriptional output generated by concurrent MAPK and Wnt activation, we performed bulk RNA-seq following stimulation with 4OHT, CHIR99021, or both ligands in combination. Combined 4OHT and CHIR99021 treatment identified 364 Wnt signaling-associated genes that were synergistically induced compared with either treatment alone (Supplementary Fig. S9). To determine whether this synergistic transcriptional program depends on TCF7, we additionally compared cells carrying TCF7-targeting sgRNAs with safe cutter control cells under combined 4OHT and CHIR99021 treatment. Across all 364 synergistically induced Wnt signaling genes, TCF7 perturbation produced a broad negative shift in expression (Fig. 4D), with 13 genes showing a significant change and 44 genes exceeding a log_2_ fold-change threshold of ±0.2, collectively indicating that TCF7 contributes to the transcriptional response elicited by combined MAPK and Wnt pathway activation (Fig. 4E). Finally, reanalysis of a public dataset [36] (GEO GSE209899) showed that EGR1 overexpression in HeLa cells not only induced TCF7 but also the bona fide Wnt target DKK1, further supporting the transcriptional EGR1–TCF7 feedback loop as a mechanism of MAPK–Wnt integration (Fig. 4F).

Collectively, our analyses reveal three principles of the RAF–MAPK transcriptional response. First, RAF1 activation induces a temporally ordered TF cascade; second, only a subset of induced TFs, most prominently EGR1 and FOS, dominate downstream regulatory control; and third, the transcriptional EGR1–TCF7 feedback loop connects RAF–MAPK signaling to Wnt-responsive transcription. These findings shift the focus from a broad list of induced genes to a compact regulatory architecture, revealing how RAF–MAPK signaling is propagated through a TF hierarchy and transcriptionally coupled to the Wnt pathway via the EGR1–TCF7 feedback loop.

## Discussion

The MAPK signaling cascade has long served as a model for how extracellular cues are translated into defined transcriptional responses. Immediate-early and delayed response programs have been described in depth [3, 6, 8, 9], yet how early transcription factors interact to orchestrate downstream programs has remained poorly understood. Here, we applied targeted Perturb-seq to systematically dissect the RAF–MAPK transcriptional network, uncovering key features of its architecture.

Several aspects of our results are consistent with well-established features of inducible MAPK transcriptional responses. In particular, rapid induction of AP-1 and EGR family transcription factors downstream of ERK signaling has been documented extensively [37], and TCF/LEF factors are established mediators of canonical Wnt transcriptional output [38]. Likewise, broad cross-talk between MAPK/ERK and Wnt/β-catenin pathways has also been described in multiple developmental and cancer contexts [39]. Kim *et al.* identified a hidden positive feedback loop caused by cross-talk between the Wnt and ERK pathways, suggesting that such feedback mechanisms can contribute to sustained activation of both pathways in cancer [40]. It has also been shown that EGR1 and TCF7L2 cooperate in a positive feedback loop that amplifies LCN2 expression and promotes aggressive cancer behaviors in esophageal squamous cell carcinoma cells [41]. In addition, aberrant activation of both the MAPK and Wnt pathways is commonly observed in other cancers such as colorectal cancer or melanoma [42, 43].

To reconstruct the network topology from our Perturb-seq data, we applied a parsimony principle, removing coherent feed-forward loops to derive the simplest model consistent with the data, similar to ARACNE-based approaches [44]. While coherent feed-forward loops are known to exist in transcriptional networks and may possess biologically significant functions [45], their removal aided in deriving a more streamlined network structure. Comparison with prior regulatory knowledge showed that part of the inferred network was supported by previously annotated interactions, whereas other edges were not present in the queried databases and therefore represent perturbation-derived candidate regulatory relationships. Our analysis confirmed prior knowledge that EGR1 and FOS are dominant hubs in the transcriptional RAF–MAPK response. Both factors control largely overlapping sets of target genes, often in an orthogonal manner. The observation that the EGR1–FOS double knockout largely resembled FOS loss suggests that FOS is epistatic to EGR1 for most targets, yet the synergistic regulation of EGR2-4 indicates a more nuanced interplay. This highlights how transcriptional networks combine dominant and redundant interactions to fine-tune cellular responses.

Moreover, this study identified the transcriptional positive feedback loop between EGR1 and TCF7. EGR1 is a canonical MAPK effector [46], while TCF7 is classically linked to Wnt/β-catenin signaling [47]. Signaling pathways often interact with each other through a process known as pathway cross-talk, which allows cancer cells to evade normal regulatory mechanisms. In the context of cancer, the MAPK pathway and the Wnt/β-catenin signaling pathway interact extensively [48]. Their mutual activation creates a direct transcriptional bridge between MAPK and Wnt pathways. Functional assays confirmed that combined MAPK and Wnt activation synergistically enhanced Wnt target gene expression, including DKK1, a well-established Wnt feedback regulator [49]. Importantly, loss of TCF7 reduced this synergistic transcriptional response, supporting a functional role for TCF7 in integrating MAPK- and Wnt-dependent gene-expression programs.

Our results indicate that the interplay between these two transcription factors provides a mechanism by which MAPK signaling can reinforce Wnt pathway activity, potentially enhancing oncogenic transcriptional programs. The identification of the transcriptional EGR1–TCF7 positive feedback loop provides a specific example of this phenomenon, where EGR1, a downstream transcription factor of the RAF–MEK–ERK MAPK pathway, and TCF7, a key component of the Wnt pathway, mutually reinforce each other’s expression. The EGR1–TCF7 circuit is supported by convergent evidence across Perturb-seq, bulk RNA-seq validation and external ChIP-seq data. Characterizing the precise chromatin-level mechanisms underlying this relationship represents a logical next step.

Taken together, these results add a new layer to MAPK–Wnt cross-talk, which has largely been studied at the receptor, kinase, or β-catenin level [48]. Here, we show that transcriptional network design itself encodes inter-pathway communication, a principle that may extend beyond the specific factors studied here to other contexts where these pathways are co-activated, such as colorectal cancer [50]. By defining the architecture of the RAF–MAPK transcriptional network and identifying the EGR1–TCF7 feedback loop as a transcriptional bridge between oncogenic pathways, this work establishes a framework for understanding how signaling diversity is encoded at the level of transcription factor networks.

## Supplementary Material

gkag718_Supplemental_Files

## Data Availability

Raw and processed transcriptome data have been deposited at GEO (https://www.ncbi.nlm.nih.gov/geo/) under the accession number GSE250559, which is the overarching superseries and includes the subseries GSE250558, GSE250533, GSE250532, and GSE333674. Data processing scripts and raw input data for the data processing scripts are available at Zenodo at the following doi: https://doi.org/10.5281/zenodo.20486852.

## References

[1] Cargnello M, Roux P P. Activation and function of the MAPKs and their substrates, the MAPK-activated protein kinases. Microbiol Mol Biol Rev. 2011;75:50–83. 10.1128/MMBR.00031-10. 21372320 PMC3063353

[2] Schulze A, Lehmann K, Jefferies H B et al. Analysis of the transcriptional program induced by Raf in epithelial cells. Genes Dev. 2001;15:981–94. 10.1101/gad.191101. 11316792 PMC312671

[3] Tullai J W, Schaffer M E, Mullenbrock S et al. Immediate-early and delayed primary response genes are distinct in function and genomic architecture. J Biol Chem. 2007;282:23981–95. 10.1074/jbc.M702044200. 17575275 PMC2039722

[4] Tullai J W, Schaffer M E, Mullenbrock S et al. Identification of transcription factor binding sites upstream of human genes regulated by the phosphatidylinositol 3-kinase and MEK/ERK signaling pathways. J Biol Chem. 2004;279:20167–77. 10.1074/jbc.M309260200. 14769801

[5] Jürchott K, Kuban R-J, Krech T et al. Identification of Y-box binding protein 1 as a core regulator of MEK/ERK pathway-dependent gene signatures in colorectal cancer cells. PLoS Genet. 2010;6:e1001231. 10.1371/journal.pgen.1001231. 21170361 PMC2996331

[6] Amit I, Citri A, Shay T et al. A module of negative feedback regulators defines growth factor signaling. Nat Genet. 2007;39:503–12. 10.1038/ng1987. 17322878

[7] Avraham R, Yarden Y. Feedback regulation of EGFR signalling: decision making by early and delayed loops. Nat Rev Mol Cell Biol. 2011;12:104–17. 10.1038/nrm3048. 21252999

[8] Legewie S, Herzel H, Westerhoff H V et al. Recurrent design patterns in the feedback regulation of the mammalian signalling network. Mol Syst Biol. 2008;4:190. 10.1038/msb.2008.29. 18463614 PMC2424294

[9] Uhlitz F, Sieber A, Wyler E et al. An immediate-late gene expression module decodes ERK signal duration. Mol Syst Biol. 2017;13:928. 10.15252/msb.20177554. 28468958 PMC5448165

[10] Kim E K, Choi E-J. Pathological roles of MAPK signaling pathways in human diseases. Biochim Biophys Acta. 2010;1802:396–405. 10.1016/j.bbadis.2009.12.009. 20079433

[11] Dhillon A S, Hagan S, Rath O et al. MAP kinase signalling pathways in cancer. Oncogene. 2007;26:3279–90. 10.1038/sj.onc.1210421. 17496922

[12] Sanchez-Vega F, Mina M, Armenia J et al. Oncogenic signaling pathways in the cancer genome atlas. Cell. 2018;173:321–337. 10.1016/j.cell.2018.03.035. 29625050 PMC6070353

[13] Hibshman P S, Der C J. The RAS signaling network and cancer. In: Rauen K. A., editors. The rasopathies: Genetic syndromes of the RAS/MAPK pathway. Cham: Springer Nature Switzerland; 2024; p.363–95. 10.1007/978-3-031-62945-7.

[14] Stelniec-Klotz I, Legewie S, Tchernitsa O et al. Reverse engineering a hierarchical regulatory network downstream of oncogenic KRAS. Mol Syst Biol. 2012;8:601. 10.1038/msb.2012.32. 22864383 PMC3421447

[15] Adamson B, Norman T M, Jost M et al. A multiplexed single-cell CRISPR screening platform enables systematic dissection of the unfolded protein response. Cell. 2016;167:1867–1882. 10.1016/j.cell.2016.11.048. 27984733 PMC5315571

[16] Dixit A, Parnas O, Li B et al. Perturb-Seq: dissecting molecular circuits with scalable single-cell RNA profiling of pooled genetic screens. Cell. 2016;167:1853–1866. 10.1016/j.cell.2016.11.038. 27984732 PMC5181115

[17] Schraivogel D, Gschwind A R, Milbank J H et al. Targeted Perturb-seq enables genome-scale genetic screens in single cells. Nat Methods. 2020;17:629–35. 10.1038/s41592-020-0837-5. 32483332 PMC7610614

[18] El Kassem G, Hillmer J, Boettcher M. Evaluation of Cas13d as a tool for genetic interaction mapping. Nat Commun. 2025;16:1631. 39952934 10.1038/s41467-025-56747-4 PMC11828948

[19] Replogle J M, Norman T M, Xu A et al. Combinatorial single-cell CRISPR screens by direct guide RNA capture and targeted sequencing. Nat Biotechnol. 2020;38:954–61. 10.1038/s41587-020-0470-y. 32231336 PMC7416462

[20] Cagnol S, Van Obberghen-Schilling E, Chambard J C. Prolonged activation of ERK1,2 induces FADD-independent caspase 8 activation and cell death. Apoptosis. 2006;11:337–46. 10.1007/s10495-006-4065-y. 16538383

[21] Samuels M L, Weber M J, Bishop J M et al. Conditional transformation of cells and rapid activation of the mitogen-activated protein kinase cascade by an estradiol-dependent human raf-1 protein kinase. Mol Cell Biol. 1993;13:6241–52. 8413224 10.1128/mcb.13.10.6241-6252.1993 PMC364683

[22] McMahon M . Steroid receptor fusion proteins for conditional activation of Raf–MEK–ERK signaling pathway. Meth Enzymol. 2001;332:401–17. 10.1016/s0076-6879(01)32218-8 11305114

[23] Doench J G, Fusi N, Sullender M et al. Optimized sgRNA design to maximize activity and minimize off-target effects of CRISPR–Cas9. Nat Biotechnol. 2016;34:184–91. 10.1038/nbt.3437. 26780180 PMC4744125

[24] Sanson K R, Hanna R E, Hegde M et al. Optimized libraries for CRISPR–Cas9 genetic screens with multiple modalities. Nat Commun. 2018;9:5416. 10.1038/s41467-018-07901-8. 30575746 PMC6303322

[25] Gibson D G, Young L, Chuang R-Y et al. Enzymatic assembly of DNA molecules up to several hundred kilobases. Nat Methods. 2009;6:343–5. 10.1038/nmeth.1318. 19363495

[26] Li W, Xu H, Xiao T et al. MAGeCK enables robust identification of essential genes from genome-scale CRISPR/Cas9 knockout screens. Genome Biol. 2014;15:554. 10.1186/s13059-014-0554-4. 25476604 PMC4290824

[27] Wessels H-H, Méndez-Mancilla A, Guo X et al. Massively parallel Cas13 screens reveal principles for guide RNA design. Nat Biotechnol. 2020;38:722–7. 10.1038/s41587-020-0456-9. 32518401 PMC7294996

[28] Griffith A L, Zheng F, McGee A V et al. Optimization of Cas12a for multiplexed genome-scale transcriptional activation. Cell Genomics. 2023;3:100387. 10.1016/j.xgen.2023.100387. 37719144 PMC10504673

[29] Hao Y, Hao S, Andersen-Nissen E et al. Integrated analysis of multimodal single-cell data. Cell. 2021;184:3573–3587. 10.1016/j.cell.2021.04.048. 34062119 PMC8238499

[30] Ahlmann-Eltze C, glmGamPoi H W. fitting Gamma-Poisson generalized linear models on single cell count data. Bioinformatics. 2021;36:5701–2. 10.1093/bioinformatics/btaa1009. 33295604 PMC8023675

[31] Concordet J-P, Haeussler M. CRISPOR: intuitive guide selection for CRISPR/Cas9 genome editing experiments and screens. Nucleic Acids Res. 2018;46:W242–5. 10.1093/nar/gky354. 29762716 PMC6030908

[32] Ran F A, Hsu P D, Wright J et al. Genome engineering using the CRISPR–Cas9 system. Nat Protoc. 2013;8:2281–308. 10.1038/nprot.2013.143. 24157548 PMC3969860

[33] Shaulian E, Karin M. AP-1 in cell proliferation and survival. Oncogene. 2001;20:2390–400. 10.1038/sj.onc.1204383. 11402335

[34] Regan J L, Schumacher D, Staudte S et al. Identification of a neural development gene expression signature in colon cancer stem cells reveals a role for EGR2 in tumorigenesis. iScience. 2022;25:104498. 10.1016/j.isci.2022.104498. 35720265 PMC9204726

[35] Kervestin S, Jacobson A. NMD: a multifaceted response to premature translational termination. Nat Rev Mol Cell Biol. 2012;13:700–12. 10.1038/nrm3454. 23072888 PMC3970730

[36] Cesana M, Tufano G, Panariello F et al. EGR1 drives cell proliferation by directly stimulating TFEB transcription in response to starvation. PLoS Biol. 2023;21:e3002034. 10.1371/journal.pbio.3002034. 36888606 PMC9994711

[37] Shan J, Dudenhausen E, Kilberg M S. Induction of early growth response gene 1 (EGR1) by endoplasmic reticulum stress is mediated by the extracellular regulated kinase (ERK) arm of the MAPK pathways. Biochim Biophys Acta. 2019;1866:371–81. 10.1016/j.bbamcr.2018.09.009. PMC6311436 30290239

[38] Liu J, Xiao Q, Xiao J et al. Wnt/β-catenin signalling: function, biological mechanisms, and therapeutic opportunities. Signal Transduct Target Ther. 2022;7:bqab203. 10.1038/s41392-021-00762-6 PMC8724284 34980884

[39] Jeong W-J, Ro E J, Choi K-Y. Interaction between Wnt/β-catenin and RAS–ERK pathways and an anti-cancer strategy via degradations of β-catenin and RAS by targeting the Wnt/β-catenin pathway. npj Precision Onc. 2018;2:5. 10.1038/s41698-018-0049-y. PMC5871897 29872723

[40] Kim D, Rath O, Kolch W et al. A hidden oncogenic positive feedback loop caused by crosstalk between Wnt and ERK pathways. Oncogene. 2007;26:4571–9. 10.1038/sj.onc.1210230. 17237813

[41] Zhao Y, Xia Q, Liu Y et al. TCF7L2 and EGR1 synergistic activation of transcription of LCN2 via an ERK1/2-dependent pathway in esophageal squamous cell carcinoma cells. Cell Signalling. 2019;55:8–16. 10.1016/j.cellsig.2018.12.007. 30557604

[42] Horst D, Chen J, Morikawa T et al. Differential WNT activity in colorectal cancer confers limited tumorigenic potential and is regulated by MAPK signaling. Cancer Res. 2012;72:1547–56. 10.1158/0008-5472.CAN-11-3222. 22318865 PMC3571091

[43] Biechele T L, Kulikauskas R M, Toroni R A et al. Wnt/β-catenin signaling and AXIN1 regulate apoptosis triggered by inhibition of the mutant kinase BRAFV600E in human melanoma. Sci Signal. 2012;5:ra3. 10.1126/scisignal.2002274. 22234612 PMC3297477

[44] Margolin A A, Nemenman I, Basso K et al. ARACNE: an algorithm for the reconstruction of gene regulatory networks in a mammalian cellular context. BMC Bioinf. 2006;7:S7. 10.1186/1471-2105-7-S1-S7. PMC1810318 16723010

[45] Milo R, Shen-Orr S, Itzkovitz S et al. Network motifs: simple building blocks of complex networks. Science. 2002;298:824–7. 10.1126/science.298.5594.824. 12399590

[46] Wang B, Guo H, Yu H et al. The role of the transcription factor EGR1 in cancer. Front Oncol. 2021;11:642547. 10.3389/fonc.2021.642547. 33842351 PMC8024650

[47] Doumpas N, Lampart F, Robinson M D et al. TCF/LEF dependent and independent transcriptional regulation of Wnt/β-catenin target genes. EMBO J. 2019;38:e98873. 10.15252/embj.201798873. 30425074 PMC6331726

[48] Zeller E, Hammer K, Kirschnick M et al. Mechanisms of RAS/β-catenin interactions. Arch Toxicol. 2013;87:611–32. 10.1007/s00204-013-1035-3. 23483189

[49] Niida A, Hiroko T, Kasai M et al. DKK1, a negative regulator of Wnt signaling, is a target of the beta-catenin/TCF pathway. Oncogene. 2004;23:8520–6. 10.1038/sj.onc.1207892. 15378020

[50] Zhan T, Ambrosi G, Wandmacher A M et al. MEK inhibitors activate Wnt signalling and induce stem cell plasticity in colorectal cancer. Nat Commun. 2019;10:2197. 10.1038/s41467-019-09898-0. 31097693 PMC6522484

