# Supplementary material for: Perturb-seq reveals TCF7 as a transcriptional link between MAPK- and Wnt-driven gene expression
Source: Nucleic Acids Res. 2026 Jul 29;54(14):gkag718. doi: 10.1093/nar/gkag718 (PMC13416742; doi:10.1093/nar/gkag718)
Supplement: gkag718_Supplemental_Files [file gkag718_supplemental_files.zip › Supplementary data legends.docx]

**Perturb-seq reveals TCF7 as a transcriptional link between MAPK- and Wnt-driven gene expression**

**AUTHORS**

Ghanem El Kassem ^1^, Anja Sieber ^2^, Bertram Klinger ^2^, Florian Uhlitz ^2^, David Steinbrecht ^2,3^, Mirjam van Bentum ^2,6^, Shawez Khan ^1^, Jasmine Hillmer ^1^, Jennifer von Schlichting ^2^, Reinhold Schäfer ^4,5^, Nils Blüthgen ^*# 2,3,5^, Michael Boettcher ^*# 1^

^1^ Institute of Molecular Medicine, Section for Molecular Medicine of Signal Transduction, Faculty of Medicine, Martin-Luther-University Halle-Wittenberg, 06120 Halle (Saale), Germany

^2^ Institute of Pathology, Charité - Universitätsmedizin Berlin, Charitéplatz 1, 10117 Berlin, Germany

^3^ Institut für Biologie, Humboldt Universität zu Berlin, Haus 18, Philippstr. 13, 10115 Berlin

^4^ Comprehensive Cancer Center, Charité - Universitätsmedizin Berlin, Charitéplatz 1, 10117 Berlin, Germany

^5^ German Consortium for Translational Cancer Research (DKTK)

^6^ Max Delbrück Center for Molecular Medicine, Robert-Rössle-Straße 10, 13125 Berlin. Germany

* To whom correspondence should be addressed. Email: [michael.boettcher@medizin.uni-halle.de](mailto:michael.boettcher@medizin.uni-halle.de). Correspondence may also be addressed to: nils.bluethgen@charite.de

**SUPPLEMENTARY DATA LEGENDS**

Supplementary Data 1: Map of pMB1-10x vector for CRISPR screen sgRNA expression.

Supplementary Table 1: sgRNA Library sgRNA sequences.

Supplementary Table 2: Proliferation screen read count table and MAGeCK MLE values.

Supplementary Table 3: Modified TAP-seq targets inner primers for gene expression library amplification.

Supplementary Table 4: sgRNA sequences used for the generation of EGR1 and FOS double knockout clonal lines.
