# Supplementary material for: Perturb-seq reveals TCF7 as a transcriptional link between MAPK- and Wnt-driven gene expression
Source: Nucleic Acids Res. 2026 Jul 29;54(14):gkag718. doi: 10.1093/nar/gkag718 (PMC13416742; doi:10.1093/nar/gkag718)
Supplement: gkag718_Supplemental_Files [file gkag718_supplemental_files.zip › Supplementary Figures.pdf]

Perturb-seq reveals TCF7 as a transcriptional link between MAPK- and Wnt-driven gene expression

AUTHORS

Ghanem El Kassem [1], Anja Sieber [2], Bertram Klinger [2], Florian Uhlitz [2], David Steinbrecht [2,3], Mirjam van Bentum [2,6], Shawez Khan [1], Jasmine Hillmer [1], Jennifer von Schlichting [2], Reinhold Schäfer [4,5], Nils Blüthgen [*# 2,3,5], Michael Boettcher [*# 1]

[1] Institute of Molecular Medicine, Section for Molecular Medicine of Signal Transduction, Faculty of Medicine, Martin-Luther-University Halle-Wittenberg, 06120 Halle (Saale), Germany

[2] Institute of Pathology, Charité - Universitätsmedizin Berlin, Charitéplatz 1, 10115 Berlin, Germany

[3] Institut für Biologie, Humboldt Universität zu Berlin, Haus 18, Philippstr. 13, 10115 Berlin

[4] Comprehensive Cancer Center, Charité - Universitätsmedizin Berlin, Charitéplatz 1, 10115 Berlin, Germany

[5] German Consortium for Translational Cancer Research (DKTK)

[6] Max Delbrück Center for Molecular Medicine, Robert-Rössle-Straße 10, 13125 Berlin. Germany

* To whom correspondence should be addressed. Email: michael.boettcher@medizin.uni-halle.de. Correspondence may also be addressed to: nils.bluethgen@charite.de

Supplementary Figures

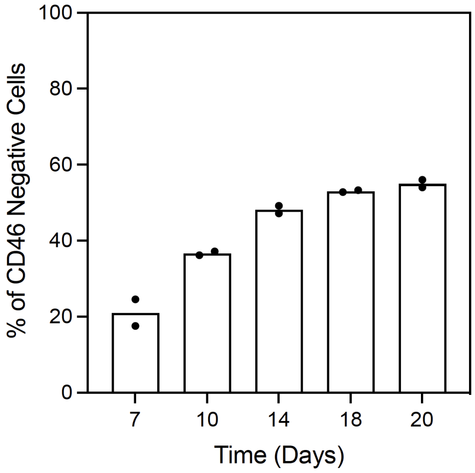

Supplementary Fig. 1 - CD46 knockout kinetics in HEK293ΔRAF1:ER cells at different time points after lentiviral infection. Percentage of CD46 knockout cells was determined via flow cytometry analysis of >10,000 cells stained with CD46 antibodies (Miltenyi). CD46 was used as a surrogate marker for perturbation efficiency because it is a ubiquitously expressed, non-essential surface protein whose loss can be quantified sensitively by flow cytometry.

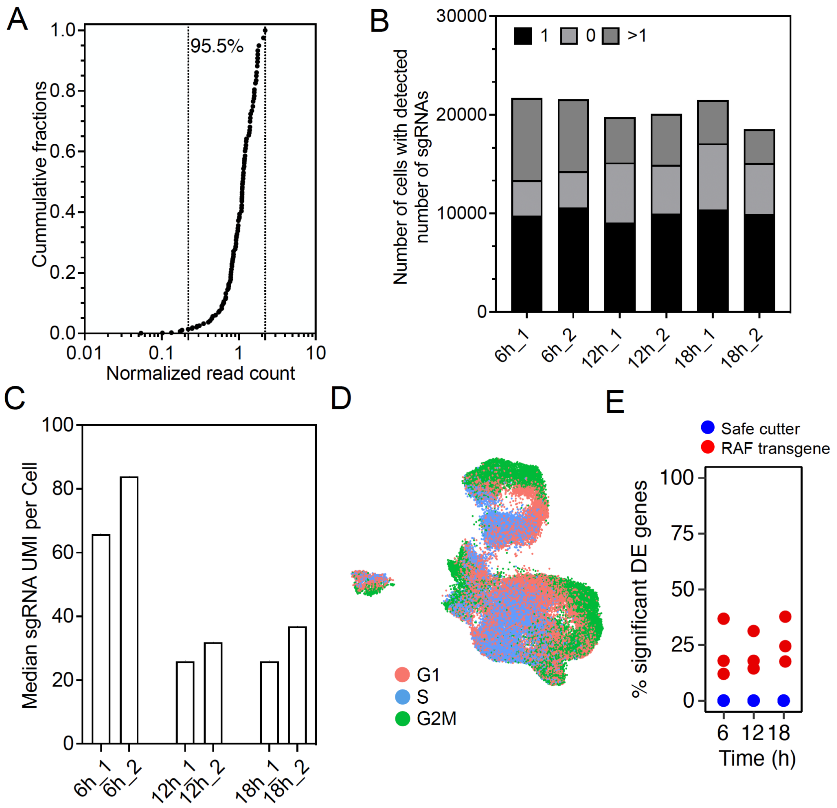

Supplementary Fig. 2 - Quality assessment of Perturb-seq screen performance. (A) Distribution of the pooled sgRNA library used for all Perturb-seq and proliferation screens. (B) Total number of recovered cells and number of cells with 0, 1, and >1 sgRNAs detected in the respective Perturb-seq samples. (C) Median sgRNA UMI counts per cell detected in the respective Perturb-seq samples. (D) Distribution of cell cycle phases G1, S, and G2M on the integrated UMAPs. (E) Percentage of significantly differentially expressed genes in the RAF1-knockout cells from the total number of genes induced by RAF activation (adjusted p-value<0.05). Red = RAF1 sgRNAs, Blue = safe cutter sgRNAs.

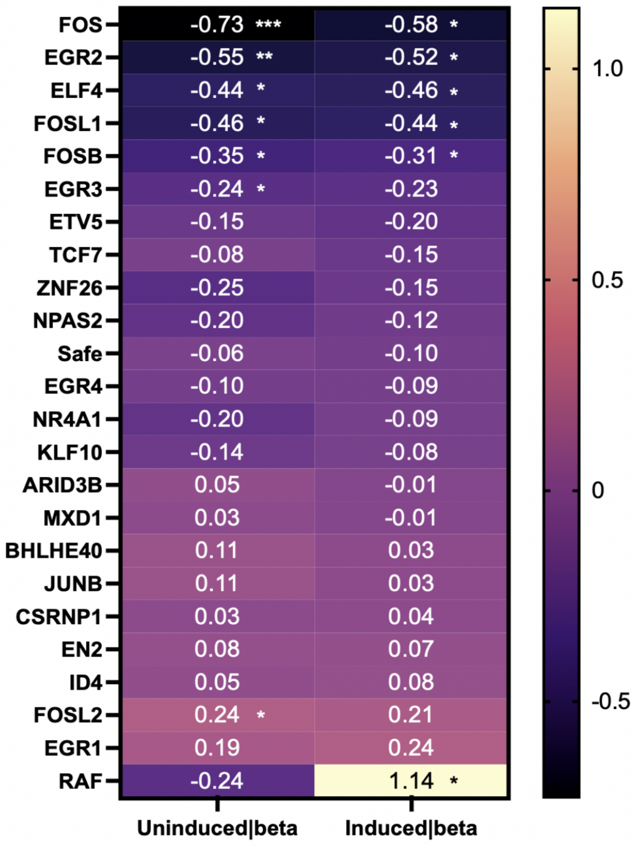

Supplementary Fig. 3 - CRISPR/Cas9 proliferation screen in HEK293ΔRAF1:ER cells. MAGeCK MLE beta scores and corresponding significance are shown. * Wald FDR <0.05, ** Wald FDR <0.01, *** Wald FDR <0.001

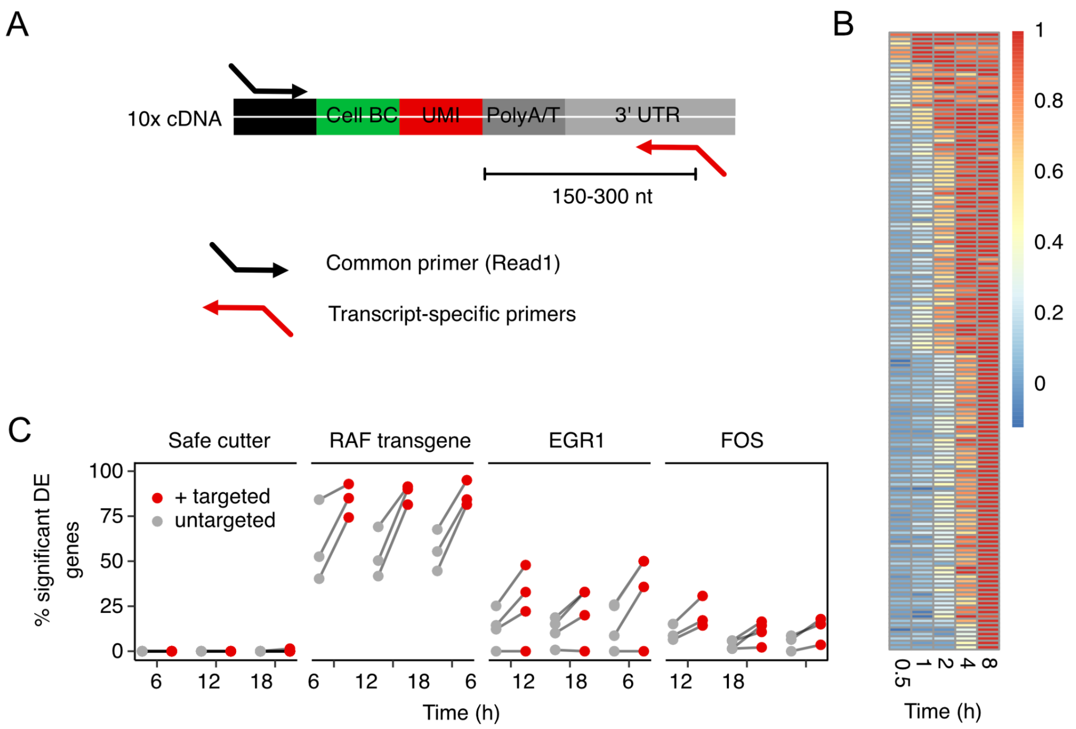

Supplementary Fig. 4 - Targeted scRNA-Seq library amplification enhances Perturb-seq sensitivity. (A) Schematic of the modified TAP-seq approach. **(B)** Selection of 140 candidate genes for modified TAP-seq. Log_2 gene expression fold changes from bulk RNA-Seq analysis of significantly induced genes after pulse induction of the RAF1 transgene for different times (FDR = 1%), normalized to gene-wise maximum Log_2 fold changes. **(C)** Difference in percentage of significant differentially expressed genes from the 140 selected genes for the modified TAP-seq between targeted and untargeted approach after knockout of RAF1 transgene, EGR1, FOS, and safe cutter control.

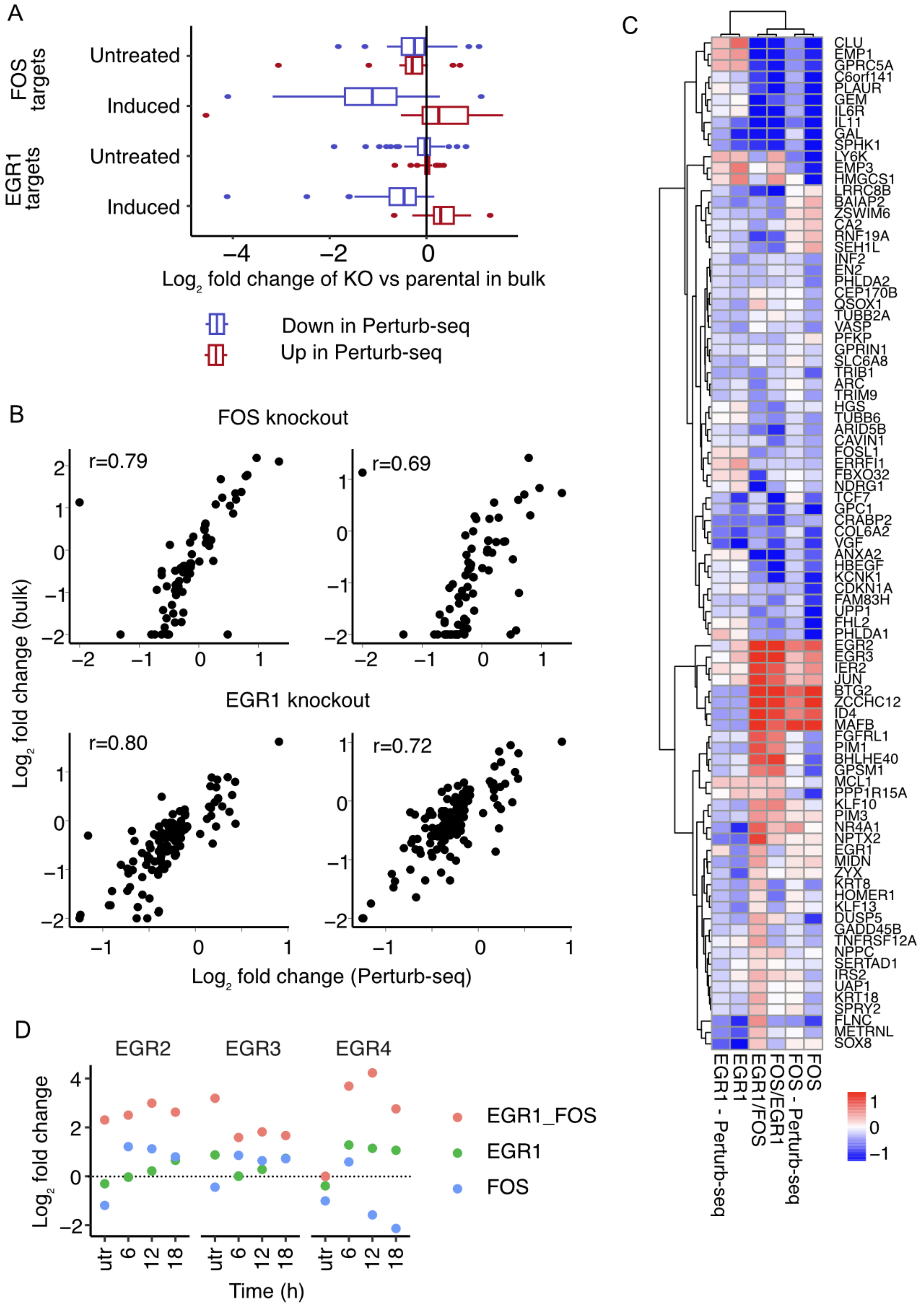
A
FOS targets
EGR1 targets
Untreated
Induced
Untreated
Induced
−4
−2
0
Log2 fold change of KO vs parental in bulk
Down in Perturb-seq
Up in Perturb-seq
B
FOS knockout
r=0.79
r=0.69
EGR1 knockout
r=0.80
r=0.72
Log2 fold change (bulk)
Log2 fold change (Perturb-seq)
C
CLU
EMP1
GPRC5A
C6orf141
PLAUR
GEM
IL6R
IL11
GAL
SPHK1
LY6K
EMP3
HMGCS1
LRRC8B
BAIAP2
ZSWIM6
CA2
RNF19A
SEH1L
INF2
EN2
PHLDA2
CEP170B
QSOX1
TUBB2A
VASP
PFKP
GPRIN1
SLC6A8
TRIB1
ARC
TRIM9
HGS
TUBB6
ARID5B
CAVIN1
FOSL1
ERRFI1
FBXO32
NDRG1
TCF7
GPC1
CRABP2
COL6A2
VGF
ANXA2
HBEGF
KCNK1
CDKN1A
FAM83H
UPP1
FHL2
PHLDA1
EGR2
EGR3
IER2
JUN
BTG2
ZCCHC12
ID4
MAFB
FGFRL1
PIM1
BHLHE40
GPSM1
MCL1
PPP1R15A
KLF10
PIM3
NR4A1
NPTX2
EGR1
MIDN
ZYX
KRT8
HOMER1
KLF13
DUSP5
GADD45B
TNFRSF12A
NPPC
SERTAD1
IRS2
UAP1
KRT18
SPRY2
FLNC
METRNL
SOX8
EGR1 - Perturb-seq
EGR1
EGR1/FOS
FOS/EGR1
FOS - Perturb-seq
FOS
D
EGR2
EGR3
EGR4
Log2 fold change
EGR1_FOS
EGR1
FOS
Time (h)

Supplementary Fig. 5 - Bulk RNA-Seq analysis of individual and combinatorial EGR1 and FOS perturbations. (A) Average $\log_2$ fold changes for genes that are identified as up- and down-regulated target genes of EGR1 and FOS in perturb-seq for bulk KO clones compared to parental clones. **(B)** Correlation between transcriptional changes detected via Perturb-seq and bulk RNA-Seq of EGR1- or FOS-perturbed cells from 2 different single knockout clonal lines of each gene. **(C)** Heatmap of transcriptional changes detected via bulk RNA-Seq from cells with individual or combinatorial EGR1 and FOS perturbations. Perturb-seq results from individual EGR1 and FOS perturbations are shown for comparison. **(D)** EGR2, EGR3, and EGR4 transcript levels determined via bulk RNA-Seq of EGR1/FOS single or double knockout clones, induced with 4OHT for 0h (utr), 6h, 12h, or 18h.

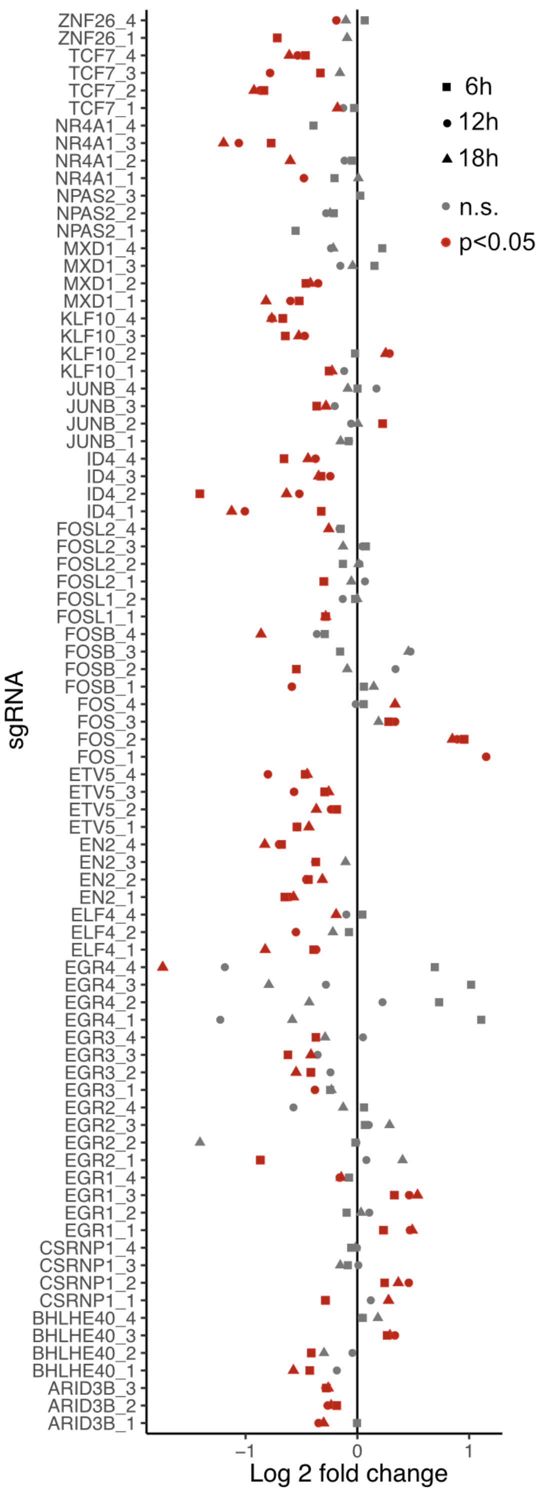

Supplementary Fig. 6 - Target gene expression changes following sgRNA perturbation. Log_2 fold change in target gene expression for individual sgRNAs at 6 h, 12 h, and 18 h after RAF1:ER activation, measured from Perturb-seq data. Red: significant changes compared to non-targeting controls ($p < 0.05$); Grey: non-significant changes.

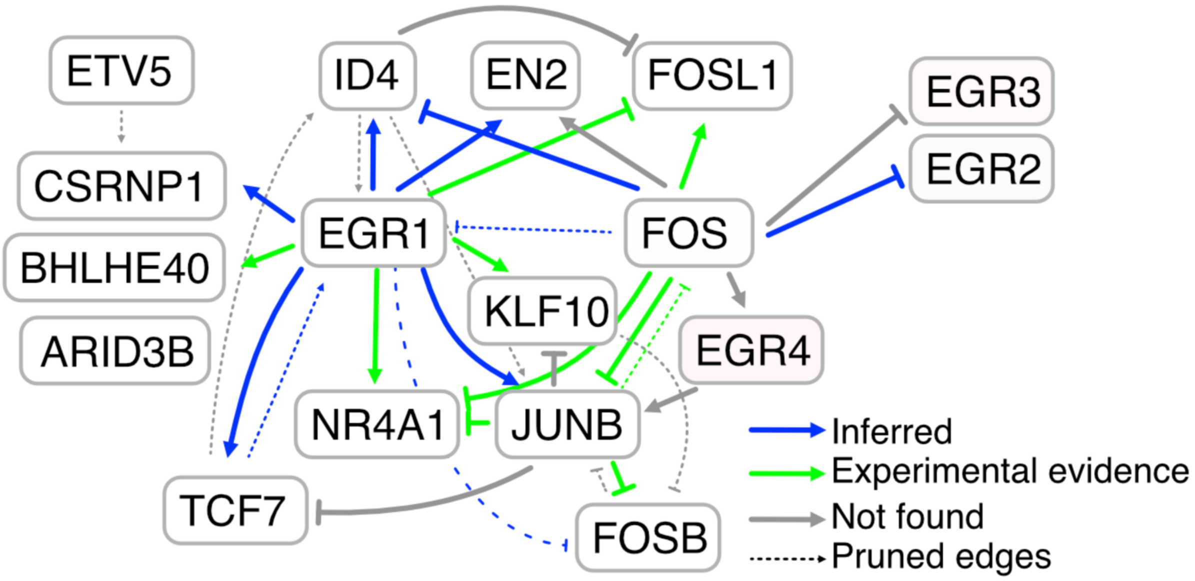

Supplementary Fig. 7 - De novo model of the TF core network highlighting interactions represented in the OmniPath database. Directional transcriptional interactions between all perturbed TFs are shown. Blue edges indicate interactions listed in OmniPath without associated literature references; green edges indicate interactions in OmniPath supported by published experimental evidence; grey edges indicate interactions not found in the OmniPath database; dotted edges indicate interactions that were pruned in our de novo transcription model in Figure 3E.

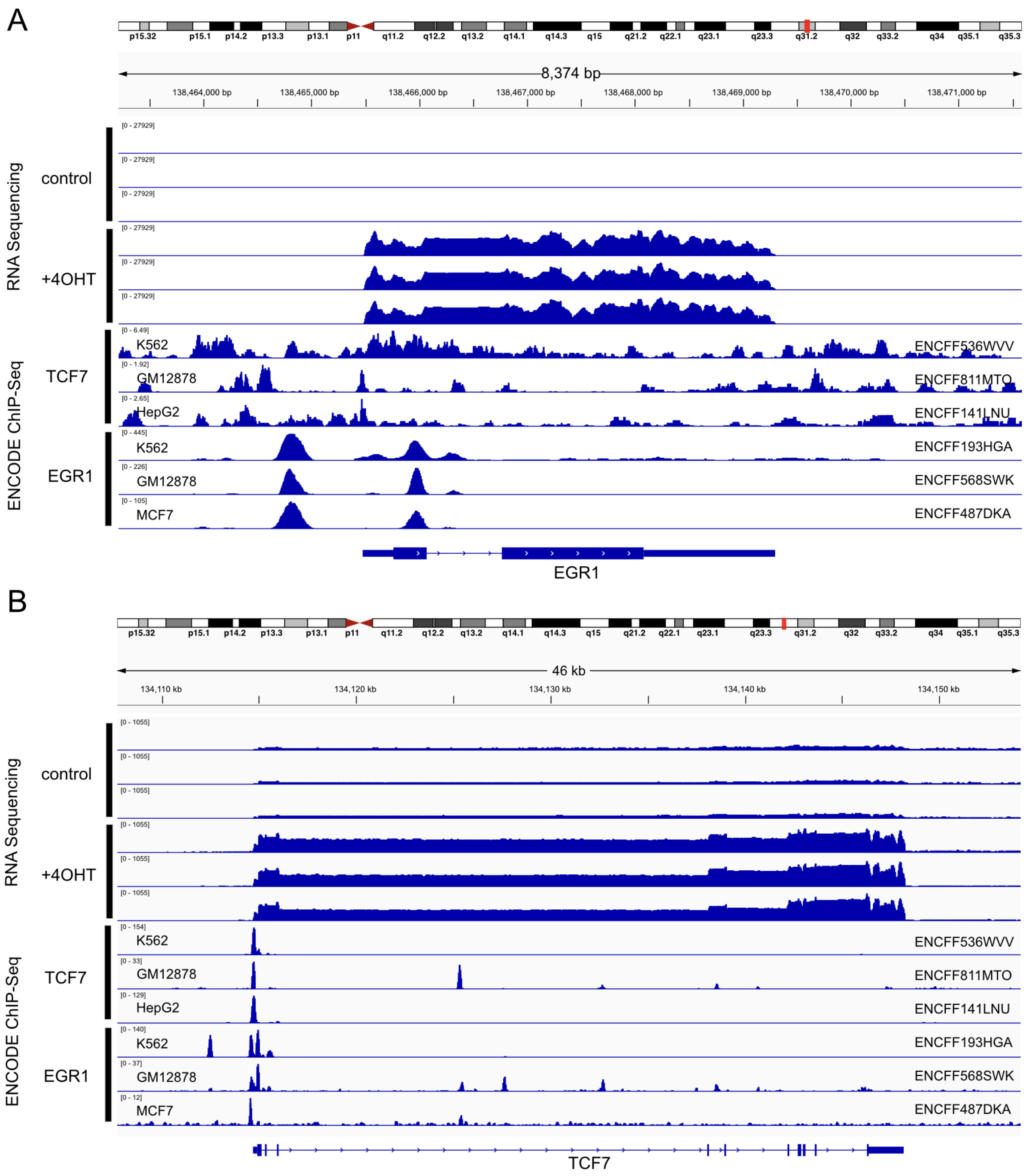

Supplementary Fig. 8 - Public ENCODE ChIP-seq tracks showing promoter-proximal binding of EGR1 and TCF7 at the EGR1 and TCF7 loci. (A) Genome browser view of the EGR1 locus showing full-length RNA-seq signal from control and 4OHT-treated HEK293ΔRAF1:ER cells, together with ENCODE ChIP-seq tracks for TCF7 and EGR1 across the indicated cell lines. **(B)** Genome browser view of the TCF7 locus showing RNA-seq signal from control and 4OHT-treated HEK293ΔRAF1:ER cells and ENCODE ChIP-seq tracks for TCF7 and EGR1. RNA-seq coverage confirms expression of TCF7 transcripts in 4OHT-treated cells.

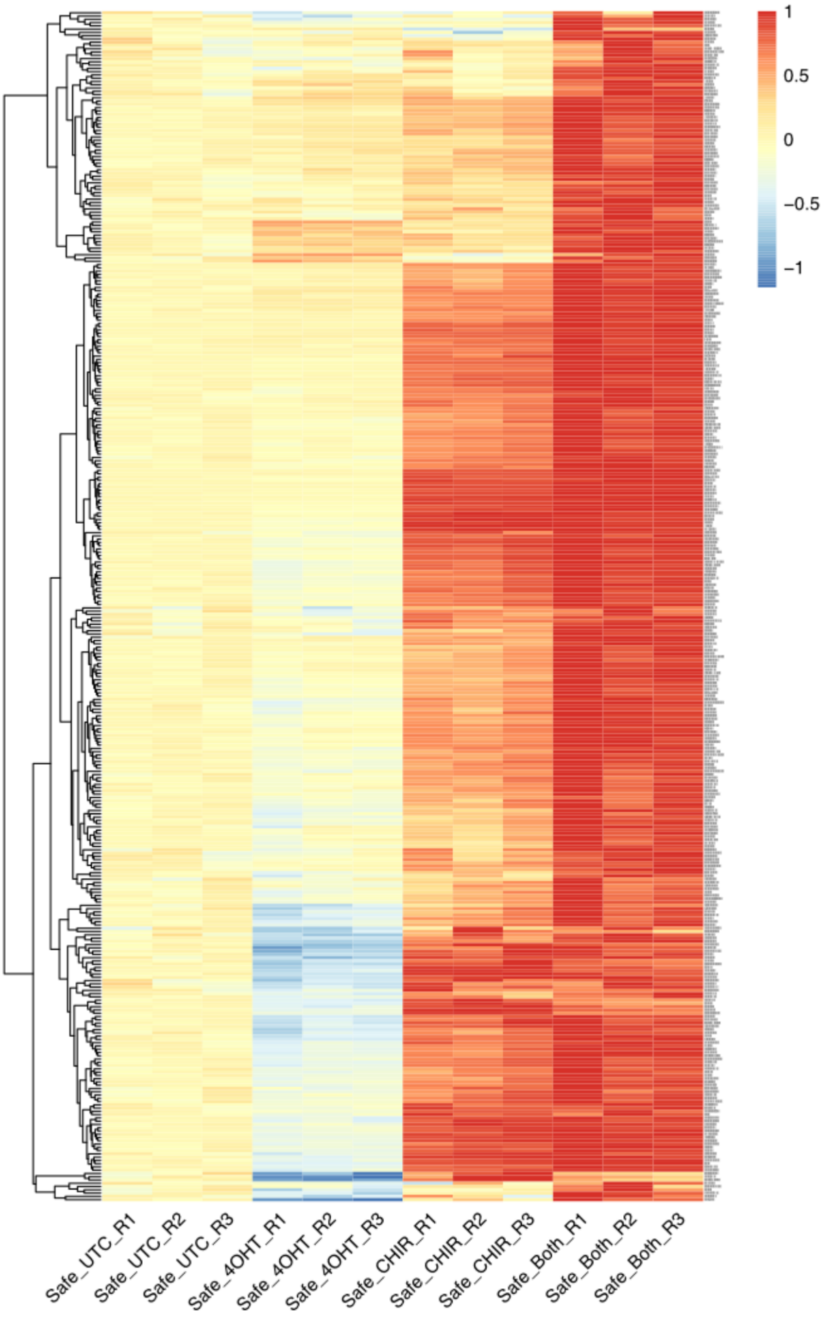

Supplementary Fig. 9 - Genes synergistically activated by combined MAPK and Wnt pathway activation. Heatmap of transcriptional changes detected via bulk RNA-Seq from cells treated with 0.5 μM 4OHT for 12 h, 10 μM CHIR99021 for 6 h, or both. Shown are the 364 Wnt signaling-associated genes identified as synergistically activated by the combined treatment. Color scale represents scaled expression values.
